# How to Put It Plainly? Findings From Two Randomized Controlled Studies on Writing Plain Language Summaries for Psychological Meta-Analyses

**DOI:** 10.3389/fpsyg.2021.771399

**Published:** 2021-12-16

**Authors:** Martin Kerwer, Marlene Stoll, Mark Jonas, Gesa Benz, Anita Chasiotis

**Affiliations:** ^1^Leibniz Institute for Psychology (ZPID), Trier, Germany; ^2^Leibniz Institute for Resilience Research (LIR), Mainz, Germany

**Keywords:** plain language summaries, science communication, lay summaries, guideline, knowledge acquisition, accessibility, understanding, empowerment

## Abstract

Plain language summaries (PLS) aim to communicate research findings to laypersons in an easily understandable manner. Despite the societal relevance of making psychological research findings available to the public, our empirical knowledge on how to write PLS of psychology studies is still scarce. In this article, we present two experimental studies investigating six characteristics of PLS for psychological meta-analyses. We specifically focused on approaches for (1) handling technical terms, (2) communicating the quality of evidence by explaining the methodological approach of meta-analyses, (3) explaining how synthesized studies operationalized their research questions, (4) handling statistical terms, (5) structuring PLS, and (6) explaining complex meta-analytic designs. To develop empirically validated guidelines on writing PLS, two randomized controlled studies including large samples stratified for education status, age, and gender (*N*_Study1_=2,288 and *N*_Study2_=2,211) were conducted. Eight PLS of meta-analyses from different areas of psychology were investigated as study materials. Main outcome variables were user experience (i.e., perceived accessibility, perceived understanding, and perceived empowerment) and knowledge acquisition, as well as understanding and knowledge of the quality of evidence. Overall, our hypotheses were partially confirmed, with our results underlining, among other things, the importance of explaining or replacing content-related technical terms (i.e., theoretical concepts) and indicating the detrimental effects of providing too many details on statistical concepts on user experience. Drawing on these and further findings, we derive five empirically well-founded rules on the lay-friendly communication of meta-analytic research findings in psychology. Implications for PLS authors and future research on PLS are discussed.

## Introduction

Insightful and well-written scientific papers are sometimes difficult to understand. Even researchers like ourselves at times encounter papers, perhaps focusing on new techniques or concepts that we are unfamiliar with, that we find hard to understand. This is more likely to occur when reading papers from disciplines outside our field of expertise – at least if these papers were not explicitly written for an interdisciplinary audience. Simply grasping the main ideas and results of this kind of paper might constitute an arduous task and could potentially hinder the interdisciplinary exchange of knowledge. If this is the case for scientists, imagine the difficulties that non-scientists (i.e., laypersons) face when they are interested in (the findings of) scientific publications with high societal or personal relevance.

Understanding science in general and especially scientific evidence published in journal articles is not easy for laypersons for multiple reasons (Bromme and Goldman, 2014). For a start, scientific articles often employ technical terms and refer to statistical concepts without explaining them to their readers (e.g., Rakedzon et al., 2017; Hauck, 2019). This procedure is undoubtedly often well-justified, as the main audience of scientific journals consists of fellow researchers who are frequently working in the same field as the authors and who have considerable theoretical and statistical expertise (this is closely linked to the notion of the “arena of internal scientific communication” by Peters, 2013). To specifically address the needs and interests of their scientific audience (and relevant gatekeepers such as editors), researchers may pay more attention to the scientific relevance of their findings and less attention to their practical relevance to the broader public when discussing their results in their publications (see also Radford, 2011; Salita, 2015). This strong focus on a scientific audience may, however, make it burdensome for non-scientists to access the current scientific literature to fulfill their need for valid and understandable information (see also Nunn and Pinfield, 2014). There are manifold relevant groups of laypersons in this context: Stakeholders (e.g., for decisions on policies), patients (for health-related decisions in medicine), practitioners, but also the interested public in general may benefit from access to research findings (Kaslow, 2015; Rodgers, 2017). For example, the current COVID-19 crisis highlighted that the public yearns – at least to some degree – for reliable scientific information and guidance (e.g., Post et al., 2021).

To close this gap (i.e., to make knowledge contained in scientific articles available to the public), plain language summaries (PLS) – easily comprehensible research summaries that complement scientific abstracts – were introduced for biomedical research (FitzGibbon et al., 2020). The pioneering role this particular area of research has taken is probably due to the high relevance that its findings have for laypeople’s everyday life (see Santesso et al., 2008). We argue that psychological findings are also of considerable interest to laypeople (see also Kaslow, 2015). Yet most guidelines on how to write PLS focus solely on biomedical research, for example, the Cochrane guidelines (Cochrane Methods, 2013; Cochrane Norway, 2016, 2019), and the scarce empirical research to validate PLS guidelines has almost exclusively taken place in the biomedical field (e.g., Santesso et al., 2015; Raynor et al., 2018; Anzinger et al., 2020; Buljan et al., 2020). Writing PLS for psychological research is thus not a straightforward task. Validated discipline-specific guidance – based on robust evidence from experimental research and taking characteristics idiosyncratic to psychology as a discipline situated at the crossroads between the humanities and the sciences into account – is practically non-existent (see also Teo, 2017). For example, how does a researcher communicate psychological concepts like “attention” or “attachment,” terms commonly used in everyday language that actually describe very sophisticated issues, views, or theories as scientific terms? Alternatively, how should meta-analytic findings that deviate considerably from the usual methodological approach in medicine be explained? This may, for instance, be the case when personality variables are correlated with indicators of well-being. The typical strategy of comparing intervention groups with each other and a control group, as used in drug effectiveness studies, does not apply here. Therefore, we argue that there is a considerable need to develop empirically validated guidelines for writing PLS in psychology. To tackle this issue, we present two randomized controlled studies investigating psychology-specific aspects of how to optimally communicate meta-analytical evidence to laypersons. Based on the findings from these studies, we subsequently provide rules on writing PLS.

In a recent systematic review of theory, guidelines and empirical research on PLS, we included a total of 90 records. These include 36 theoretical articles primarily discussing the rationale behind and the aims of PLS, 21 guidance-related articles and 33 articles reporting empirical studies (Stoll et al., 2021). Based on extracted information from these records, we developed a conceptual framework of the aims, characteristics, criteria, and outcomes investigated in PLS research. The aims of PLS (e.g., to impart knowledge about scientific findings to laypersons) determine the characteristics taken into account when composing PLS (e.g., the kind of words that are used). PLS criteria outlined in guidelines are specifications of such characteristics (e.g., the PLS should not be written in jargon). The effectiveness of such criteria, in turn, is evaluated by their impact on predefined outcomes constituting measureable operationalizations of the aims (e.g., knowledge tests). We found that the aims of PLS mentioned, discussed or investigated in the literature could be classified into six categories: accessibility, understanding, knowledge, empowerment, communication of research, and improvement of research. In the current studies, we focus on the first four aims because they target the individual PLS user level, whereas improvement and communication of research aims relate to (the relationship between) society and academia at a higher level. Accessibility, understanding and empowerment directly relate to a subjective user’s perspective and can, therefore, also be described as aims that refer to user experience, as user experience research focuses primarily on the attractiveness, understandability, and usefulness of a product or medium (e.g., Buchanan, 1992; Morville, 2004; Rosenbaum, 2010). Outcomes measuring user experience aims of PLS in experimental studies comprise subjective statements and self-reports on perceived readability of a PLS (accessibility), perceived ease of comprehending the meaning of a PLS on a content level (understanding) and/or usefulness of a PLS for making decisions (empowerment). Knowledge can be measured using (objective) knowledge tests (e.g., Alderdice et al., 2016; Buljan et al., 2018; Kerwer et al., 2021).

In our review, we identified several shortcomings of the empirical research on PLS carried out so far. Of the 33 empirical articles, we found only eight (24%) contrasted different versions of PLS, whereby only six of them (18%) used an experimental research design to do so (Santesso et al., 2015; Alderdice et al., 2016; Kirkpatrick et al., 2017; Buljan et al., 2020; Silvagnoli et al., 2020). Many empirical studies on the effectiveness of PLS, however, either compared PLS to different summary formats (e.g., Buljan et al., 2018) or only evaluated one single PLS (e.g., Santesso et al., 2015), making it hard to infer the suitability of certain characteristics of a PLS from the observed outcomes or to generalize study results. Furthermore, most empirical studies on PLS employed rather small samples that were far from representative for the general public. Participants were often highly educated (e.g., students, Alderdice et al., 2016, Kerwer et al., 2021; or healthcare professionals, Opiyo et al., 2013; Maguire and Clarke, 2014). The empirical evidence that PLS are effective for the public in general – outside narrow settings of professionals or student samples – is therefore still scarce. Research on PLS needs to address this issue by collecting data of larger and more diverse groups. This is especially important since PLS, for example, when accompanying scientific abstracts in journals, sometimes do not even aim to address a specific target group but are rather directed toward anyone who might be interested in psychological research or wants to know something about the topic at hand. To allow PLS to fulfill their purpose of educating and empowering the public, we thus need to investigate their performance in a heterogeneous readership, as well as how reader characteristics affect this performance (specifically, individual differences in interest in psychological research).

Against the background of an ongoing replication crisis in psychology (Open Science Collaboration, 2015; Świątkowski and Dompnier, 2017; Klein et al., 2018), we have argued that PLS may be a powerful tool for rebuilding public trust in psychological science, and first experimental findings (Kerwer et al., 2021) seem to support this notion (see also Carvalho et al., 2019 for non-experimental findings that point in the same direction). Because meta-analyses possess a higher quality of evidence and are instrumental for summarizing findings and guiding practitioners (Borenstein et al., 2009; Bastian et al., 2010), we further argue that the lay-friendly communication of psychological meta-analytical findings is of particular importance – even though legitimate criticisms on the validity of their findings may exist (e.g., Sharpe and Poets, 2020; Burgard et al., 2021; Patall, 2021).

When it comes to communicating meta-analytic psychological evidence to a broad audience *via* PLS, several aspects quite specific to psychological research or especially relevant in the context of psychological research exist (e.g., the vocabulary or the operationalization of psychological constructs in primary studies). Our reasoning for choosing such aspects here does not arise from the notion that they are especially difficult to explain in the context of psychology or even that they serve a unique function in this field. Rather, according to Shneider (2009), use of specific language (technical terms describing subject matter) as well as methodology (e.g., operationalization) constitute the core characteristics of a scientific discipline, which distinguish it from others. In psychology, for example, the necessity of operationalizing latent psychological constructs is distinct from other disciplines. Other aspects exist that are highly relevant in the context of psychological research – but not discipline-specific by nature – and for which empirical evidence beyond biomedical research is lacking (e.g., research designs and effect size measures in the social sciences). More specifically, in the life sciences, laypeople might be unfamiliar with the jargon typically used in scientific publications due to the fact that researchers use scientific names for species or illnesses uncommon in everyday language. In this case, technical terms need to be “translated” for some readers. For example, a plain language translation of the title “Salvage systemic therapy for advanced gastric and oesophago-gastric junction adenocarcinoma” is “Which treatments work best for advanced stomach cancer that has not responded to standard chemotherapy?” (Tomita et al., 2020). In psychological research, however, this problem is complex in another way, since the vocabulary of psychological science often does not differ from everyday language (e.g., the word “attachment,” see above). The underlying meaning might differ, however, or these words might represent very specific ideas. Without prior knowledge, laypersons, for example, might not understand what developmental psychologists mean when they are referring to “secure attachment.” In guidelines on writing PLS in medicine (Cochrane Methods, 2013; Cochrane Norway, 2016, 2019) and other disciplines (AGU, n.d.; People and Nature, n.d.), typical approaches for handling technical terms are: (1) Replacing the technical terms by non-technical terms within the text of the PLS. Transferred to the context of psychological PLS, this means that instead of using technical terms, these terms are circumscribed by using everyday language (e.g., “feeling safe in relationships” instead of “secure attachment”) and this simplified description is used consistently throughout the PLS text (*replacement*); (2) using technical terms within the text but providing an additional glossary on technical terms in which these terms are explained (*glossary*). In our research, we wanted to compare these two approaches to simply ignoring the problem, that is, to using technical terms within the text of the PLS without any explanation (*no explanation*). In hypotheses 1 and 2, we assumed that both replacing technical terms with non-technical terms and providing a glossary on technical terms would improve the user experience (perceived accessibility, perceived understanding, and perceived empowerment) and knowledge acquisition of laypeople:

*Hypothesis 1: Technical terms: glossary vs. no explanation*: Accessibility (H1a), understanding (H1b), content-related knowledge (H1c), and empowerment (H1d) are higher when a glossary explaining technical terms is provided (*glossary*) compared to leaving technical terms unexplained (*no explanation*).

*Hypothesis 2: Technical terms: replacement vs. no explanation*: Accessibility (H2a), understanding (H2b), content-related knowledge (H2c), and empowerment (H2d) are higher when technical terms are replaced by non-technical terms within the text of the PLS (*replacement*) compared to using technical terms without explanation (*no explanation*).

Second, we have argued above that meta-analytic research findings constitute an important source of information for laypeople and that this is especially true for psychology in times of the replication crisis. However, communicating meta-analytic evidence in a lay-friendly manner can be challenging. Most laypersons will likely not know that meta-analytical findings draw on multiple studies and therefore have a higher level of evidence quality than individual research findings based on one single study. Thus, to provide laypersons the opportunity to grasp the quality of evidence more accurately when evaluating a meta-analysis, they need to understand that the presented evidence does not stem from a single individual study (see Cochrane Norway, 2019). We argue that laypersons’ further understanding is improved by providing them with a lay-friendly explanation of the methodological approach underlying meta-analytic research. In fact, very current and therefore non-peer reviewed research suggests that this approach (i.e., an explanatory statement) allowed non-scientists to take the quality of evidence of preprints compared to peer-reviewed articles into account when it comes to perceived credibility (Wingen et al., 2021). In hypothesis 3, we assumed that the communication of quality of evidence by an explanation of the methodological approach affects laypersons’ knowledge on the quality of evidence (i.e., factual knowledge on the concept “meta-analysis”) and the understanding of the quality of evidence (taking this knowledge adequately into account in a transfer task).

*Hypothesis 3: Quality of evidence communication: statement vs. no statement*: Understanding of the quality of evidence (H3a) and knowledge on the quality of evidence (H3b) are higher when an explanation of the methodological approach “meta-analysis” (quality of evidence *statement*) is given compared to a condition in which no such explanation is given (*no statement*).

Third, exactly how psychological studies investigate their research questions is often not self-evident – at least to laypersons – thereby establishing a certain need to “translate psychological science” (see Kaslow, 2015). For example, how is arithmetic competence in very young infants that cannot even speak studied? How are anxiety or resilience measured? One might argue that providing laypersons with (exemplary) information on how the individual studies included in a meta-analysis (i.e., the synthesized studies) operationalized their research questions will substantially improve their understanding of what was actually analyzed in the meta-analysis and their ability to draw conclusions based on its findings. In line with this reasoning, Hoogeveen et al. (2020, p. 269) showed that laypeople were able to rate the likelihood of successful replications when “a short description of the research question, its operationalization, and the key finding” were provided in plain language. However, one might also argue that presenting this additional information may increase task-related cognitive load (e.g., Sweller et al., 2011) and therefore impede laypersons’ user experience and knowledge acquisition regarding the findings of the meta-analysis. To shed some light onto how providing information on the operationalization of the research question in the synthesized studies affects the perception of PLS (i.e., user experience) as well as the knowledge acquired through them, we examined effects of providing laypersons with this information. More specifically, we varied whether or not a subsection on the operationalization of the research question in the individual studies was included in the PLS (information on the operationalization *provided* vs. *not provided*).

*Hypothesis 4: Operationalization: subsection vs. no subsection*. Providing information on how the research question was operationalized in the synthesized studies affects the following outcome variables: Accessibility (H4a), understanding (H4b), content-related knowledge (H4c), and empowerment (H4d).

Hypotheses 1–4 were tested in a first empirical study (Study 1). Hypotheses 5–8 were tested in a second empirical study (Study 2) and partly built upon results of Study 1. For example, participants repeatedly asked for more information about what “effect size” means, how effect sizes are computed and how to interpret their values after being provided with a qualitative statement and an effect size estimate in Study 1. Against this background, the fourth aspect addressed by our research, albeit not completely psychology-specific, becomes especially relevant for psychological meta-analyses. Laypeople are most likely not familiar with the statistical concepts and especially effect size measures employed in scientific publications. In spite of the fact that first studies on communicating statistical evidence to non-scientists *via* PLS exist – especially for Cochrane PLS (e.g., Glenton et al., 2010; Santesso et al., 2015; Buljan et al., 2020) – broad consensus on this issue is still lacking in relevant guidelines. For example, Cochrane (Cochrane Methods, 2013; Cochrane Norway, 2016, 2019) states that statistical terms need to be explained if included. For this purpose, Cochrane offers specific guidelines on how to report descriptive statistics as well as standardized statements for reporting effect sizes. Other guidelines recommend that researchers remove all statistics (e.g., American Psychological Association, 2018) or do not provide any guidance on this issue (see Stoll et al., 2021 for a comprehensive review). This illustrates the need for further research in this area. We compared four approaches for explaining statistical terms and effect sizes in psychological meta-analyses: (1) the effect size is not reported – instead, a qualitative statement on the interpretation of its size is provided, e.g., “The relationship was medium-sized.” (*qualitative statement*); (2) the effect size is reported and a qualitative statement on the interpretation of its size is provided, e.g., “The correlation was *r*=0.40. This is a medium-sized relationship.” (*effect size+qualitative statement*); (3) no qualitative statement is provided, only the effect size is reported in the text and an explanation of the effect size, its boundaries and cut-off values at assist interpretation are provided below the PLS in a glossary, e.g., “What is *r* and what value of *r* implies a large effect?” (*effect size+glossary*); (4) the effect size, a qualitative statement and a glossary are provided (*effect size+glossary+qualitative statement*). In hypotheses 5 and 6, based on user feedback of Study 1, we propose that providing qualitative statements in the text of a PLS *and* a glossary on statistical terms promotes user experience and knowledge acquisition:

*Hypothesis 5*^1^*: Statistical terms: incremental effect glossary*: Accessibility (H5a), understanding (H5b), content-related knowledge (H5c), and empowerment (H5d) are higher when an effect size is reported *and* a glossary that explains statistical terms is provided after the PLS *and* a qualitative statement on the interpretation of the effect size is provided within the text of the PLS (*effect size+glossary+qualitative statement*) compared to reporting the effect size *and* providing a qualitative statement *without* a glossary (*effect size+qualitative statement*).

*Hypothesis 6^1^: Statistical terms: incremental effect qualitative statement*: Accessibility (H6a), understanding (H6b), content-related knowledge (H6c), and empowerment (H6d) are higher when an effect size is reported *and* a glossary explaining statistical terms is provided after the PLS *and* a qualitative statement on the interpretation of the effect size is provided within the text of the PLS (*effect size+glossary+qualitative statement*) compared to reporting the effect size *and* providing a glossary *without* a qualitative statement (*effect size+glossary*).

The fifth aspect, we investigated is not psychology-specific but applies to PLS in general. Cochrane guidelines (Cochrane Methods, 2013; Cochrane Norway, 2016, 2019) and existing evidence on research summary characteristics clearly point toward subheadings as an important formal element that should be included in research summaries and PLS (e.g., Kerwer et al., 2021 for PLS; but see also Hartley, 2004, 2014; Hartley and Betts, 2007 for structured abstracts in general). Additionally, first evidence suggests that further structuring the text of PLS (e.g., by means of bullet points) improves the user experience for information recipients (e.g., Ellen et al., 2014; Raynor et al., 2018; Anzinger et al., 2020). To test if these findings can be transferred to PLS of psychological meta-analyses (and can also be replicated on a conceptual level in a considerably larger and more representative sample in a randomized controlled experimental design), we compared *structured* text with formatted text blocks (e.g., by bullet points) to *unstructured* text without further formatting of text blocks within subsections. More specifically, we made the following assumptions in hypothesis 7:

*Hypothesis 7^1^: Structuring: structured vs. unstructured*: Accessibility (H7a), understanding (H7b), content-related knowledge (H7c), and empowerment (H7d) are higher when the text is *structured* compared to a condition, where no structuring is provided (*unstructured*).

The rationale for hypothesis 8 was derived from our experience testing hypotheses 1 and 2 in Study 1. We found that our participants faced problems with regard to knowledge acquisition and user experience when confronted with PLS that reported complex meta-analytical designs (i.e., results of a meta-analytic structural equation model). One might argue that meta-analytic structural equation models belong to the most complex meta-analytic techniques in the current literature. Still, we had to concede that the statement explaining the methodological approach of meta-analyses (i.e., the quality of evidence statement; see hypothesis 3 and Supplementary Material 1) solely focused on an overly simple type of “univariate” meta-analytical design (i.e., a design testing whether one effect is different from zero in the literature). This type of statement might not promote laypersons’ understanding of more complex meta-analytic designs. This is especially important because an integral feature of (even simple) meta-analytic designs is that they can model heterogeneity in effect sizes, and, as a consequence, estimate moderator effects (e.g., Sánchez-Meca and Marín-Martínez, 2010). Simple meta-analytical designs including moderator effects are arguably still not very complex compared to structural equation modeling approaches (Steinmetz et al., 2020). They are, however, significantly more complex than the design presented in our statement to explain the meta-analytic approach in hypothesis 3. Drawing on this notion, we chose this “slightly more complex” meta-analytical design (i.e., moderator analyses) as a starting point for investigating how to support PLS readers in their understanding of more complex meta-analytic design types. For this purpose, we extended the quality of evidence statement, we used for examining hypothesis 3 to outline moderator analyses. The following hypothesis was specified for comparing this *extended* quality of evidence statement compared to the *regular*, “unextended” quality of evidence statement that was used to test hypothesis 3:^2^

*Hypothesis 8^1^: Quality of evidence communication: regular statement vs. extended statement*: Explaining complex meta-analytic designs affects the following outcome variables: Accessibility (H8a), understanding (H8b), content-related knowledge (H8c), and empowerment (H8d).

Hypotheses 1–4 and hypotheses 5–8 were preregistered at PsychArchives. Hypotheses 5–8 were preregistered after results regarding hypotheses 1–4 were available but before data collection for Study 2 began.

## Study 1

In our first study, we investigated the following PLS characteristics: approaches for explaining technical terms, approaches for communicating the quality of evidence, and approaches for communicating how the research question was operationalized in the synthesized studies. Study procedures, as well as the original German items and full texts of the PLS, were preregistered at PsychArchives.^3^ English translations of items are reported in the manuscript and exemplary English translations of PLS, the quality of evidence statement as well as knowledge items are provided in Supplementary Material 1.

### Materials and Methods

#### Sample

We recruited a large general population sample (*N*_Target_=2,004, see *Sample Size Calculation and Power Analysis*) *via* the panel provider Respondi. More specifically, Respondi was tasked with the recruitment and monetary compensation of study participants. The following specific quota conditions were specified: Age (18–44, 45, or older), gender (men, women), and secondary school education level (lower track: “Hauptschulabschluss,” middle track: “Mittlere Reife,” and higher track: “Hochschulreife”). As there were 2×2×3=12 overall combinations of those quota conditions (e.g., male participants with “Mittlere Reife” and younger than 45years), the target sample size for each of these combinations was 2,004/12=167. Additionally, the following inclusion criteria applied: Participants (1) had to possess German language skills at native speaker level, (2) had to have successfully graduated from secondary school, and (3) were not currently studying psychology or holding a degree in psychology.

Because all participants were allowed to complete started sessions, the final sample size was slightly larger than planned: *N*=2,038 participants completed the questionnaire and *N*=2,288 responded to at least one outcome variable (two PLS were presented to each participant, and some participants dropped out after answering outcome variables on the first PLS). The number of complete observations for each combination of quota conditions ranged from 165 to 174. On average, participants were 46.31years old (*Mdn*=45, minimum=18, maximum=90, *SD*=14.94), and our sample contained slightly more women (50.48%) than men. Moreover, the proportion of participants with lower educational status was slightly higher compared to the other educational status groups (31.73% Hochschulreife, 32.74% Mittlere Reife, and 35.53% Hauptschulabschluss). Our specific aim guiding sample selection was to investigate PLS performance of a heterogeneous readership. The multivariate distribution of demographic variables, however, is not intended to be representative of the German population.

#### Design

There were two study arms. Study arm A was designed for testing the effects of different approaches for explaining technical terms of psychological core concepts (hypotheses 1 and 2). In study arm B, effects of providing information on the operationalization of the research question in the synthesized studies were tested (hypothesis 4). Hypothesis 3, on the effect of (not) providing a statement on the quality of evidence (i.e., on the methodological approach of meta-analyses) was tested in both study arms. Participants either received or did not receive a statement on the methodological approach underlying meta-analyses (i.e., quality of evidence statement).

In study arm A, participants were randomly assigned to one of the three approaches for explaining technical terms (replacement, glossary, and no explanation). Within each condition, all participants read two PLS, each summarizing a different meta-analysis from resilience research (based on meta-analyses by Rasmussen et al., 2019, PLS_RR1, and Groth et al., 2019, PLS_RR2). The same approach for explaining technical terms was applied across both PLS read by each participant. No information on operationalization was provided for these PLS.

In study arm B, participants were randomly assigned to one of the two conditions for providing information on the operationalization of the research question in the synthesized studies (information provided, information not provided). Within each condition, all participants read two PLS, each summarizing a different meta-analyses from research on infant development (based on meta-analyses by Christodoulou et al., 2017, PLS_ID1, and Dunst et al., 2012, PLS_ID2). In both PLS presented to test hypothesis 4, technical terms were consistently replaced by non-technical terms.

Table 1 illustrates the design and conditions of Study 1. As can be seen in Table 1, Study 1 had a between-subjects design with three factors and – because two of these factors were varied separately in different study arms (see above) – there were 10 (3×2+2×2) conditions overall.

**Table 1:** Study 1 design. Outline of experimental conditions.

Condition	Study arm	PLS	Quality of evidence communication	Information on operationalization in synthesized studies	Approach for explaining technical terms
1	Resilience research (A)	PLS_RR1/PLS_RR2	No statement	Not provided	Replacement
2	Resilience research (A)	PLS_RR1/PLS_RR2	Statement	Not provided	Replacement
3	Resilience research (A)	PLS_RR1/PLS_RR2	No statement	Not provided	Glossary
4	Resilience research (A)	PLS_RR1/PLS_RR2	Statement	Not provided	Glossary
5	Resilience research (A)	PLS_RR1/PLS_RR2	No statement	Not provided	No explanation
6	Resilience research (A)	PLS_RR1/PLS_RR2	Statement	Not provided	No explanation
7	Infant development (B)	PLS_ID1/PLS_ID2	No statement	Provided	Replacement
8	Infant development (B)	PLS_ID1/PLS_ID2	Statement	Provided	Replacement
9	Infant development (B)	PLS_ID1/PLS_ID2	No statement	Not provided	Replacement
10	Infant development (B)	PLS_ID1/PLS_ID2	Statement	Not provided	Replacement

PLS, plain language summary; PLS_ID1=PLS of Christodoulou et al., 2017; PLS_ID2=PLS of Dunst et al., 2012; PLS_RR1=PLS of Rasmussen et al., 2019; and PLS_RR2=PLS of Groth et al., 2019.

#### Procedure

The study was conducted online using the survey software Unipark. The online questionnaire was created and hosted by the authors. After being invited by Respondi, participants completed an informed consent form. Thereafter, inclusion criteria were checked and participants were assigned to one of the 10 experimental conditions of our study. Two PLS were presented to each participant. The design and content of these PLS were dependent on the assigned experimental condition. Participants read each of these two PLS for at least 3min and answered the outcome measures on (perceived) accessibility, (perceived) understanding, understanding of the quality of evidence, and (perceived) empowerment on the same webpage. After reading the PLS, knowledge items were presented on the following page. Finally, participants completed a short user survey and were redirected to Respondi for monetary compensation. All study procedures were approved by the ethics committee of Trier University. Participants spent a mean duration of approximately 18min on completing the questionnaire.

#### Variables

Information on the following outcomes was assessed on the same page as the corresponding PLS for confirmatory analyses: (perceived) accessibility (“The language in this text is easy to read.”), (perceived) understanding (“The information in this text is understandable.”), and (perceived) empowerment (“After reading this text, I can participate in a discussion about the topic.”). We measured responses to these items on Likert scales ranging from 1 (*fully disagree*) to 8 (*fully agree*).

Understanding of the quality of evidence was measured *via* a simple decision task as the preference of meta-analytic evidence over the evidence of an individual study. More specifically, after participants read the PLS, we stated that results of a fictitious new study contradicted the result of the meta-analysis the participants had just read (“Imagine you are talking to a friend about the topic of the review. Your friend knows of a slightly more recent study that contradicts the result of the review.”). Thereafter, we asked them to decide which study result they perceived as more trustworthy (“Which result would you trust?” 1=“The result of the slightly more recent study,” 8=“The result of the review”). Therefore, higher values on this semantic differential indicated that participants correctly understood the higher level of quality of evidence represented in a meta-analysis.

PLS-specific knowledge items were administered on a separate page after each PLS: Content-related knowledge on the key message of the PLS, and PLS-specific knowledge of the quality of evidence referring to what the researchers did (i.e., that the results of X studies were summarized in the review) were assessed *via* one forced-choice format item with four options. After PLS-specific knowledge items on the second PLS were presented, knowledge of the quality of evidence in general (i.e., what a meta-analysis is) was assessed *via* one forced-choice format item with six options. Responses on all knowledge items were recoded to correct (“1”) or incorrect (“0”) for statistical analyses.

A full list of exploratory outcomes and further potential covariates that were collected is included in the preregistration of Study 1 materials. In this article, we present exploratory analyses regarding the covariate interest in psychological research (“I am interested in psychological research.”). Agreement to this statement was assessed on an eight-point Likert scale.

#### Statistical Analysis

##### Sample Size Calculation and Power Analysis

We conducted a power analysis using the software GPower (Faul et al., 2009). As a statistical test, we selected F-tests on between-level factors in repeated measures ANOVAs. The following parameters were specified: Small (*f*=0.10) effect size, alpha=0.05, power=0.90 with three groups, two measurements and an expected correlation between measures of 0.50. This power analysis indicated that a sample size of 954 participants was required. Thus, we decided to recruit at least 1,000 participants for each study arm. Because additional quota restrictions applied, our total target sample size was 2,004 participants.

##### Statistical Model

We analyzed our data in R (R Core Team, 2021) by means of mixed models (to draw on their flexibility compared to simple repeated measures ANOVAs) using the lme4 package (Bates et al., 2015). Using mixed models allowed us, for example, to include incomplete data of participants that rated only one PLS (and not both). For inferential statistics and group comparisons, the packages lmertest (Kuznetsova et al., 2017) and multcomp (Hothorn et al., 2008) were employed.^4^ As indicated above, hypotheses 1 and 2 were tested in the subsample of participants in study arm A and hypothesis 4 in the subsample of participants in study arm B. Hypothesis 3 was tested based on the whole sample. Our hypotheses were tested based on the significance of regression coefficients of dummy-coded variables. In study arm A, the reference category for dummy variables was *PLS_RR1* with *no explanation* on technical terms and *no statement* on the quality of evidence (i.e., condition 5 in Table 1). In study arm B, the reference category was *PLS_ID2* with no information on operationalization in synthesized studies (*not provided*) and *no statement* on the quality of evidence (i.e., condition 9 in Table 1). One-sided hypothesis tests were conducted when appropriate, and the significance of effects was tested at *p*<0.05. To obtain effect size estimates for individual fixed effects, we computed partial R-squared statistics as a measure of incrementally explained variance using the r2glmm package (Jaeger et al., 2017). Additionally, marginal and conditional R-squared statistics for mixed models were calculated (Nakagawa et al., 2013; as implemented in the R package MuMIN; Barton, 2020). Finally, data were discarded if participants failed to respond to any confirmatory outcome (i.e., dropped out prior to providing a response, 599 participants) or if participants completed the survey more than once (in this case, only data from the first completion were analyzed, 13 participants). We provide information on the final analysis-specific number of participants and observations (i.e., rated PLS) in the relevant tables on confirmatory analyses.

### Results

Descriptive statistics on confirmatory outcomes from Study 1 are provided in Table 2. Accessibility, understanding, and empowerment were highly correlated (all *r*s>0.56), which is why we will report results of the corresponding confirmatory analyses in an aggregated manner within the text. Mixed model results on Study 1, separated by outcome variable, are provided in Tables 3 and 4.

**Table 2:** Study 1 descriptive statistics (means and SDs) of confirmatory outcomes separated by design condition.

Outcome	Approach for explaining technical terms			Information on operationalization in synthesized studies		Quality of evidence communication	
	Replacement			Glossary			No explanation			Provided			Not provided			Statement			No statement		
	*M*	*SD*	*n*	*M*	*SD*	*n*	*M*	*SD*	*n*	*M*	*SD*	*n*	*M*	*SD*	*n*	*M*	*SD*	*n*	*M*	*SD*	*n*
Accessibility	5.219	2.105	726	5.129	2.055	643	4.692	2.147	695	5.907	1.996	1,119	6.251	1.774	1,143	5.527	2.036	2,100	5.614	2.107	2,226
Understanding	5.290	2.012	727	5.355	1.956	643	4.616	2.091	695	5.909	1.954	1,119	6.245	1.759	1,139	5.536	1.971	2,101	5.666	2.058	2,222
Empowerment	4.315	2.079	726	4.304	2.021	642	3.651	2.025	696	4.734	2.024	1,120	4.982	1.989	1,140	4.472	2.037	2,009	4.509	2.106	2,225
Understanding of the quality of evidence	4.788	1.705	717	5.043	1.634	632	4.569	1.745	692	4.716	1.891	1,118	4.671	1.854	1,117	4.850	1.818	2,078	4.637	1.769	2,198

*M*=arithmetic mean; *SD*=standard deviation; and *n*=number of observations.

**Table 3:** Study 1 results of confirmatory analyses for user experience outcomes: accessibility, understanding, and empowerment.

Study arm A						Study arm B					
Outcome	Parameter	EST	SE	*p*	*R*^2^ Beta	Outcome	Parameter	EST	SE	*p*	*R*^2^ Beta
Accessibility *N*_obs_=2,064 *N*_ID_=1,101	Random effect (participant) variance	2.675				Accessibility *N*_obs_=2,262 *N*_ID_=1,183	Random effect (participant) variance	2.610			
	Residual variance	1.673					Residual variance	0.983			
	Intercept 1	5.010	0.118	<0.001			Intercept 2	6.341	0.091	<0.001	
	Replacement	0.545	0.138	<0.001	0.012		Operationalization provided	−0.338	0.103	0.001	0.008
	Glossary	0.437	0.142	0.002	0.007		Quality of evidence statement	−0.100	0.103	0.334	0.001
	Glossary – replacement	−0.108	0.141	0.442							
	Quality of evidence statement	−0.092	0.115	0.421	0.000						
	PLS_RR2	−0.550	0.058	<0.001	0.017		PLS_ID1	−0.113	0.042	0.008	0.001
	Marginal/conditional *R*^2^	0.030/0.627					Marginal/conditional *R*^2^	0.009/0.729			
Understanding *N*_obs_=2,065 *N*_ID_=1,101	Random effect (participant) variance	2.476				Understanding *N*_obs_=2,258 *N*_ID_=1,184	Random effect (participant) variance	2.268			
	Residual variance	1.536					Residual variance	1.198			
	Intercept 1	4.950	0.113	<0.001			Intercept 2	6.389	0.089	<0.001	
	Replacement	0.683	0.133	<0.001	0.020		Operationalization provided	−0.320	0.099	0.001	0.007
	Glossary	0.751	0.137	<0.001	0.022						
	Glossary – replacement	0.068	0.135	0.613							
	Quality of evidence statement	−0.136	0.110	0.217	0.001		Quality of evidence statement	−0.122	0.099	0.221	0.001
	PLS_RR2	−0.544	0.056	<0.001	0.018		PLS_ID1	−0.205	0.047	<0.001	0.003
	Marginal/conditional *R*^2^	0.046/0.635					Marginal/conditional *R*^2^	0.011/0.658			
Empowerment *N*_obs_=2,064 *N*_ID_=1,101	Random effect (participant) variance	2.824				Empowerment *N*_obs_=2,260 *N*_ID_=1,184	Random effect (participant) variance	2.814			
	Residual variance	1.315					Residual variance	1.225			
	Intercept 1	3.853	0.116	<0.001			Intercept 2	5.110	0.097	<0.001	
	Replacement	0.680	0.137	<0.001	0.019		Operationalization provided	−0.220	0.108	0.043	0.003
	Glossary	0.664	0.141	<0.001	0.017						
	Glossary – replacement	−0.016	0.140	0.911							
	Quality of evidence statement	−0.015	0.114	0.899	0.000		Quality of evidence statement	−0.049	0.108	0.654	0.000
	PLS_RR2	−0.431	0.052	<0.001	0.011		PLS_ID1	−0.275	0.047	<0.001	0.005
	Marginal/conditional *R*^2^	0.034/0.693					Marginal/conditional *R*^2^	0.008/0.699			

Estimates are based on mixed models with (contrasts of) fixed effects for independent variables/PLS and random effects for participants. Separate models were estimated per outcome for each study arm. EST, estimates for variances of residuals and random effects, unstandardized regression weights, and marginal/conditional R-squares (Nakagawa et al., 2013); SE, standard error, *p*=*p*-value of two-tailed significance test (please note that some tests of hypotheses were one-tailed); PLS, plain language summary; *N*_obs_=number of rated PLS; *N*_ID_=number of participants who rated at least one PLS, Intercept 1=no explanation, no quality of evidence statement, PLS_RR1; Intercept 2=no information on operationalization, no quality of evidence statement, PLS_ID2; PLS_ID1=PLS of Christodoulou et al., 2017; PLS_ID2=PLS of Dunst et al., 2012; PLS_RR1=PLS of Rasmussen et al., 2019; and PLS_RR2=PLS of Groth et al., 2019.

**Table 4:** Study 1 results of confirmatory analyses for knowledge acquisition, PLS-specific knowledge of the quality of evidence and knowledge on the quality of evidence in general.

Outcome	Parameter	EST	SE	*p*	OR
Content-knowledge (Study arm A) *N*_obs_=1,990 *N*_ID_=1,026	Random effect (participant) variance	1.617			
	Intercept 1	1.415	0.164	<0.001	4.117
	Replacement	0.328	0.168	0.051	1.388
	Glossary	−0.107	0.171	0.532	0.899
	Glossary – replacement	−0.434	0.172	0.012	0.648
	Quality of evidence statement	−0.082	0.138	0.552	0.921
	PLS_RR2	−1.179	0.124	<0.001	0.308
Content-knowledge (Study arm B) *N*_obs_=2,230 *N*_ID_=1,147	Random effect (participant) variance	0.762			
	Intercept 2	1.213	0.119	<0.001	3.363
	Operationalization	0.117	0.111	0.293	1.124
	Quality of evidence statement	−0.067	0.111	0.548	0.935
	PLS_ID1	−0.994	0.106	<0.001	0.370
(PLS-specific) knowledge of the quality of evidence *N*_obs_=4,420 *N*_ID_=2,173	Random effect (participant) variance	1.164			
	Intercept 3	−0.754	0.091	<0.001	0.470
	Quality of evidence statement	0.548	0.087	<0.001	1.729
	PLS_ID1	−0.156	0.099	0.113	0.855
	PLS_RR1	0.086	0.111	0.437	1.090
	PLS_RR2	0.053	0.111	0.636	1.054
Knowledge on the quality of evidence in general *N*_ID_=2,044	Intercept 4	0.527	0.064	<0.001	1.694
	Quality of evidence statement	0.253	0.094	0.0068	1.288

Estimates are based on logistic mixed models with (contrasts of) fixed effects for independent variables/PLS and random effects for participants. Separate models were estimated for each study arm for content-knowledge. For PLS-specific knowledge of the quality of evidence and knowledge on the quality of evidence in general, one model for both study arms was estimated. EST, estimates for variances of random effects, unstandardized regression weights; SE, standard error, *p*=*p*-value of two-tailed significance test (please note that some tests of hypotheses were one-tailed); OR, odds ratio; PLS, plain language summary. *N*_obs_=number of rated PLS; *N*_ID_=number of participants who rated at least one PLS, Intercept 1=no explanation, no quality of evidence statement, PLS_RR1; Intercept 2=no information on operationalization, no quality of evidence statement, PLS_ID2, Intercept 3=no quality of evidence statement, PLS_ID2, Intercept 4=no quality of evidence statement; PLS_ID1=PLS of Christodoulou et al., 2017; PLS_ID2=PLS of Dunst et al., 2012; PLS_RR1=PLS of Rasmussen et al., 2019; and PLS_RR2=PLS of Groth et al., 2019.

#### H1 and H2 Approach for Explaining Technical Terms

Overall, accessibility, understanding, and empowerment ratings as well as knowledge acquisition were lower for PLS_RR2 compared to PLS_RR1 (see inferential statistics in Table 3).

##### Accessibility, Understanding, and Empowerment

**Figure 1:**
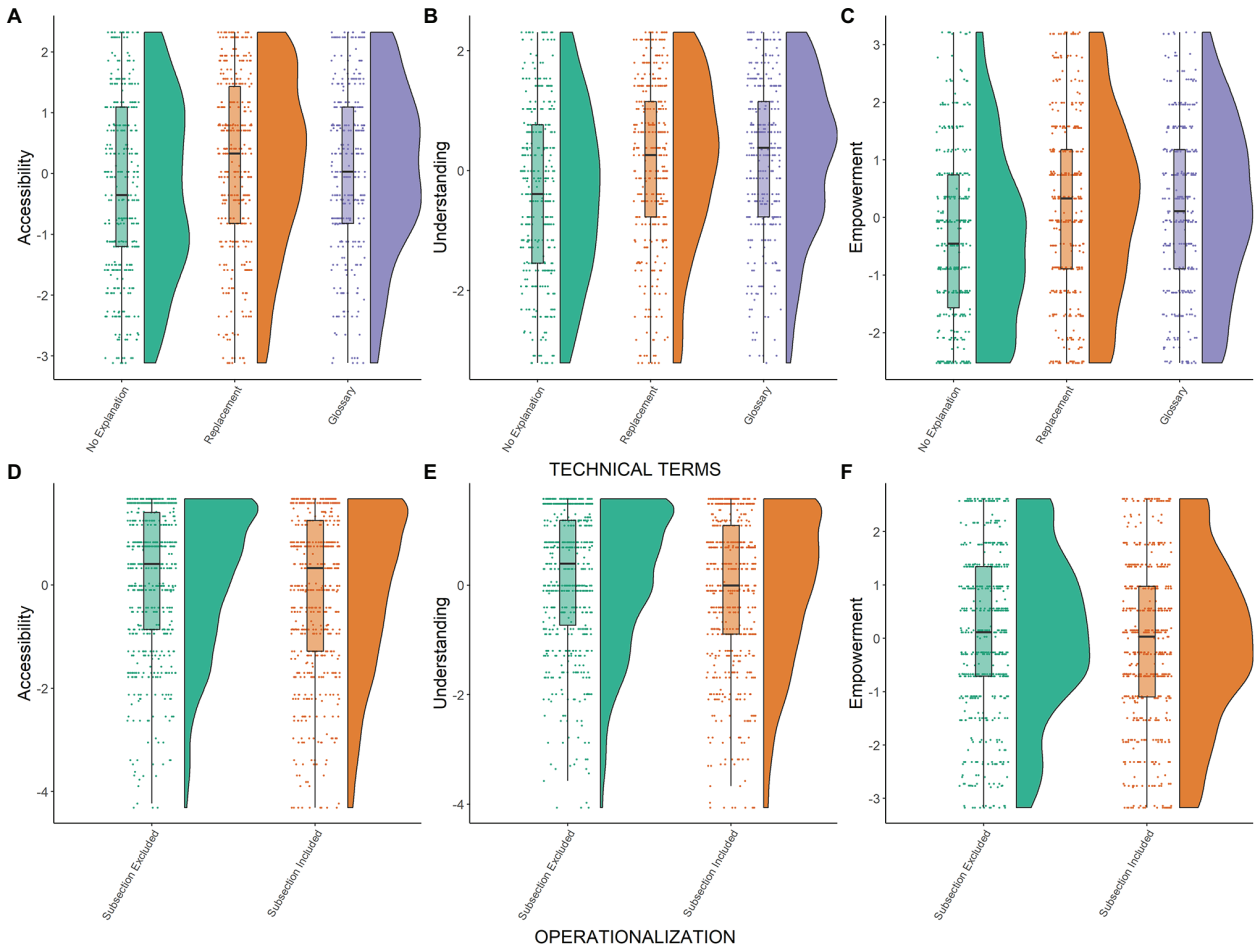
Results study 1. Raincloud plots for user experience outcomes: accessibility **(A,D)**, understanding **(B,E)**, and empowerment **(C,F)**. Residual scores are depicted separated by experimental conditions: Technical terms **(A–C)**, operationalization **(D,E)**. Residual scores were obtained from a mixed model that controlled for participant as random factor and for presented plain language summaries (PLS)/quality of evidence statement as fixed factors.

Mean values for accessibility and understanding indicated that participants tended to agree to the corresponding statements (mean values higher than 4.50). More specifically, mean values on accessibility and understanding ranged from 4.62 to 5.36 (on a 1–8 scale) across experimental conditions. Descriptively, mean values for empowerment were, however, lower and ranged from 3.65 to 4.32 (see Table 2). Perceived accessibility, understanding, and empowerment of the PLS were significantly higher in the *replacement* or *glossary* condition compared to the *no explanation* condition (see Table 3; Figures 1A–C). Thus, H1a, H1b, H1d, and H2a, H2b and H2d were fully confirmed. All differences between the *replacement* and *glossary* conditions were non-significant on user experience outcome measures (see Table 3).

##### Content-Related Knowledge

Descriptively, the proportion of correct answers was higher in the *replacement* condition (68.84%), compared to the *no explanation* condition (63.60%) or the *glossary* condition (62.03%). Inferential analyses revealed that the likelihood of answering content-related knowledge items correctly was significantly higher in the *replacement* condition compared to the other conditions (i.e., *no explanation* and *glossary*), whereas the difference between providing *no explanation* and providing a *glossary* was non-significant (see Table 4). Consequently, H2c was confirmed and H1c was rejected.

#### H3 Quality of Evidence Statement

##### Understanding of the Quality of Evidence, User Experience, and Content-Related Knowledge

**Figure 2:**
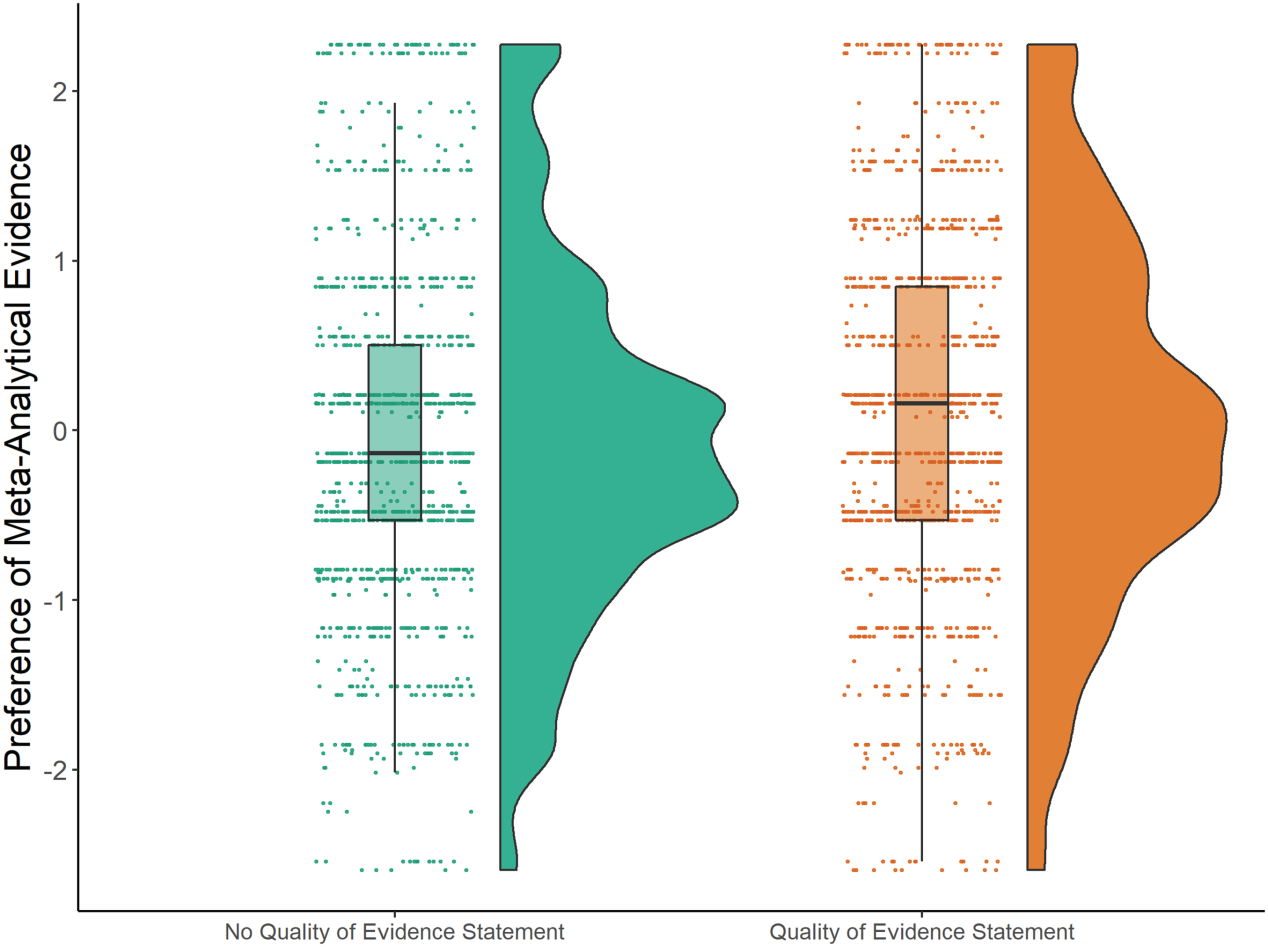
Results study 1. Raincloud plots for understanding of the quality of evidence as a preference of meta-analytic evidence in a decision task. Residual scores are depicted separated by experimental conditions: statement on the quality of evidence presented vs. no statement on the quality of evidence presented. Residual scores were obtained from a mixed model that controlled for participant/presented PLS as random factors.

Overall, mean values for preferring meta-analytic evidence over evidence from single studies in our decision task (indicating a better understanding of the quality of evidence) were lower in the *no statement* on the quality of evidence condition (*M*=4.64) compared to the *statement* condition (*M*=4.85, on a 1–8 semantic differential, with 1 indicating preference of the single study and 8 indicating preference of meta-analytic evidence). This difference was statistically significant. The preference of meta-analytical evidence significantly increased when a statement on the quality of evidence explaining the methodological approach underlying meta-analyses had been presented (*b*=0.22, *SE*=0.07, *p*<0.001, see also Figure 2). Consequently, H3a is confirmed. There were no significant effects of the quality of evidence statement on user experience outcome measures or content-related knowledge (all *p*s>0.068, see Supplementary Material 2).

##### Knowledge on the Quality of Evidence in General and PLS-Specific

For PLS-specific knowledge of the quality of evidence (i.e., what the researchers did) and knowledge on the quality of evidence in general (i.e., what a meta-analysis is), we found significant effects for our statement on the methodological approach underlying meta-analyses (see Table 4). Participants were more likely to correctly respond to the PLS-specific quality of evidence knowledge items (45.86 vs. 35.50%) and were more likely to provide the correct answer to a general knowledge item on the methodological approach of meta-analyses (68.57 vs. 62.88%, see Table 4). Thus, H3b was fully confirmed.

#### H4 Information on Operationalization in Synthesized Studies

Overall, perceived accessibility, understanding, and empowerment ratings as well as knowledge acquisition were lower for PLS_ID1 compared to PLS_ID2 (see Table 3).

##### Accessibility, Understanding, and Empowerment

Mean values for accessibility, understanding, and empowerment were lower when information on the operationalization of the research question was *provided* in the PLS compared to when it was *not provided* (see Table 2). Inferential analyses indicate that the corresponding differences were significant (see Table 3; and Figures 1D–F), thus confirming H4a, H4b, and H4d.

##### Content-Related Knowledge

Descriptively, the proportion of correct answers was slightly higher when information on the operationalization was *provided* (65.79%), compared to the condition providing no such information (63.64%). The difference between both conditions was, however, far from statistical significance (see Table 4). H4c is therefore rejected.

#### Exploratory Analysis: Interest in Psychological Research

In exploratory analyses, we found that interest in psychological research generally seemed to compensate – at least to some extent – for the negative effects of providing additional information on the operationalization of the research question reported above. More specifically, a positive linear effect of interest in psychological research existed for all outcomes that were related to user experience (Accessibility: *b*=0.51, *SE*=0.05, *p*<0.001, *R*^2^=0.073, Understanding: *b*=0.55, *SE*=0.05, *p*<0.001, *R*^2^=0.086, and Empowerment: *b*=0.71, *SE*=0.05, *p*<0.001, *R*^2^=0.125). In a separate model, we also tested whether effects of providing additional information on the operationalization differed depending on interest in psychological research. Our data appear to tentatively suggest that interest in psychological research might moderate the effects of providing information on the operationalization. However, the corresponding interaction was not consistently significant (Accessibility: *b*=0.27, *SE*=0.10, *p*=0.006, *R*^2^=0.005, Understanding: *b*=0.18, *SE*=0.09, *p*=0.054, *R*^2^=0.003, and Empowerment: *b*=0.18, *SE*=0.10, *p*=0.074, *R*^2^=0.002). Linear effects of interest in psychological research were also found for PLS of resilience research. Detailed results of all analyses on interest in psychological research are provided in Supplementary Material 2.

### Interim Discussion

Our results on approaches for explaining technical terms (hypotheses 1 and 2) suggest that both approaches tested for handling technical terms – replacing technical terms in the text and explaining technical terms in a glossary – seem to work satisfactorily with respect to user experience (perceived accessibility, understanding, and empowerment). However, replacing technical terms instead of retaining them in the text or explaining them *via* a glossary appears to have some beneficial effects on knowledge acquisition. Some technical issues notwithstanding (see General Discussion on technical limitations of implementing glossaries), we decided to pursue the replacement approach in Study 2.

Our analyses on hypothesis 3 show that our quality of evidence statement (i.e., the explanation of the methodological approach underlying meta-analyses) worked as intended: Providing this explanation improved our subjects’ understanding and knowledge on the quality of evidence. Further analyses suggest that the statement had no “side effects” (i.e., no statistically significant negative effects on any content-related knowledge or user experience measures); thus, we propose providing this kind of statement alongside PLS of psychological meta-analyses to improve laypersons’ understanding of the quality of evidence and assist them in their decision making.

In our tests of hypothesis 4, we found no evidence in favor of including information on the operationalization of the synthesized studies. Including this subsection seemed to have negative effects on user experience and did not affect knowledge acquisition. On the one hand, the latter finding is encouraging because it illustrates that knowledge on the key message of the PLS did not suffer when providing additional textual information on the operationalization of the research question. On the other hand, indisputable user experience losses were found, which is why we opted to not include this information in Study 2. However, it should be noted that negative effects of adding this information might be less pronounced for participants that are highly interested in psychological research (see also General Discussion on cognitive load theory and writing PLS).

We also found that, in each study arm, the two PLS presented to participants differed considerably in user experience and knowledge acquisition. We argue that this finding points toward the *complexity* of PLS as an important PLS feature. We suggest that PLS might differ in their complexity for various reasons. They might display complexity because the design of the summarized meta-analyses is complex, and they strive to communicate this in detail. This might have been the case, for example, with PLS_RR2 of study of Groth et al. (2019), which reported mediator effects in meta-analytic structural equation models. PLS may also be more complex because they strive to explain the complex theoretical background of a meta-analysis. An example for this might be PLS_ID1 of study of Christodoulou et al. (2017). This study searched the literature for evidence on an effect initially found by another author. By describing this background, our PLS might have introduced a “replicability” meta-level. In contrast, comparably simple meta-analytic designs might focus on one specific effect size (e.g., one treatment effect or correlation). We further suggest that the complexity of PLS also increases if meta-analyses report a large number of effect sizes (e.g., various simple correlation coefficients) and the PLS aims to convey this information to the reader. Against this background, negative effects of providing additional information on the operationalization may also be interpretable as a result of the increased PLS complexity due to the inclusion of additional content. We further pursued this issue of PLS complexity in Study 2.

Finally, results of our exploratory analyses illustrated that interest has to be taken into consideration as an important covariate when analyzing laypeople’s perception of PLS. It is necessary to keep in mind that the target audience ultimately reading PLS will likely be more interested in psychological research than the general public. For this reason, we opted to only include participants in Study 2 who reported a certain degree of interest in psychological research. This was done to test our PLS in a sample more similar to the target audience of PLS.

## Study 2

In our second study, we investigated the following PLS characteristics: approaches for explaining statistical terms, approaches for communicating complex meta-analytic designs *via* an extended quality of evidence statement, and approaches for structuring PLS. Study procedures, as well as the original German items and full texts of the PLS, were preregistered at PsychArchives.^5^ English translations of items are reported in the manuscript and exemplary English translations of PLS, the extended quality of evidence statement as well as knowledge items are provided in Supplementary Material 1.

### Materials and Methods

#### Sample

As in Study 1, we recruited a general population sample (*N*_Target_=2,004) *via* Respondi. Quota conditions were the same as in Study 1, and all inclusion criteria of Study 1 also applied to Study 2. Additionally, to obtain a sample that was more similar to the natural target audience of PLS, participants had to indicate that they were interested in psychological research (Item: “I am interested in psychological research.”). The inclusion criterion was an agreement of “4” or higher on an eight-point Likert scale. The choice of this specific cut-off value was based upon the lowest medium value observed for this item across all quota condition combinations of Study 1.

Once again, the final sample size was slightly larger than planned: *N*=2,078 participants completed the questionnaire and *N*=2,211 participants responded to at least one confirmatory outcome variable. Despite best efforts, the target sample size was not completely achieved for one quota condition combination, namely men who were younger than 45years of age with a Hauptschulabschluss. However, this group still reached about 90% (150 out of 167) of its intended size. The number of complete observations for each quota condition combination ranged from 150 to 194. Our participants were, on average, 46.10years old (*Mdn*=45, minimum=18, maximum=83, *SD*=15.14) and our sample contained slightly more women (50.47%) than men. Education status was evenly distributed (33.92% Hochschulreife, 32.56% Mittlere Reife, and 33.51% Hauptschulabschluss).

#### Design

The results of Study 1 pointed toward the complexity of PLS as an important design factor. In our second study, we therefore strived to examine to what extent can effects be generalized to more or less complex PLS. For this purpose, Study 2 also had two study arms. In study arm A, two PLS with comparably low complexity were presented: One PLS reported only one correlation coefficient as an effect size (based on a meta-analysis by Schwalm et al., 2021, PLS_LC1), and one PLS reported only one mean difference as an effect size (based on a meta-analysis by Bucher et al., 2020; in the ZPID’s PsychOpen CAMA system, see Burgard et al., 2021, PLS_LC2). Study arm B included two entirely different PLS with comparably high complexity: One PLS reported meta-moderator analyses (based on a meta-analysis by Bergmann and Cristia, 2016, PLS_HC1), and one PLS reported nine correlation coefficients (based on a meta-analysis by Yule et al., 2019, PLS_HC2).

As can be seen in Table 5, Study 2 had a between-subjects design with three fully crossed factors: Communication of complex meta-analytic designs by an extended quality of evidence statement, approach for explaining statistical terms and formal text structuring. Each participant was randomly assigned to one study arm and read its two corresponding PLS. Both PLS were randomly assigned to one condition (i.e., combination of the different levels of the three independent variables depicted in Table 5): one of the four approaches for explaining statistical terms, one of the two approaches for communicating complex meta-analytic designs and one of the two approaches for formal text structuring. The assigned condition was the same for both PLS. As a result, there were 16 (4×2×2) conditions in total, identical within each study arm (see Table 5).

**Table 5:** Study 2 design. Outline of experimental conditions.

Condition	Study arm	PLS	Quality of evidence communication	Structuring	Approach for explaining statistical terms
1	Low complexity (A)	PLS_LC1/PLS_LC2	Regular statement	Structured	Qualitative statement
2	Low complexity (A)	PLS_LC1/PLS_LC2	Regular statement	Unstructured	Qualitative statement
3	Low complexity (A)	PLS_LC1/PLS_LC2	Extended statement	Structured	Qualitative statement
4	Low complexity (A)	PLS_LC1/PLS_LC2	Extended statement	Unstructured	Qualitative statement
5	Low complexity (A)	PLS_LC1/PLS_LC2	Regular statement	Structured	Effect size+qualitative statement
6	Low complexity (A)	PLS_LC1/PLS_LC2	Regular statement	Unstructured	Effect size+qualitative statement
7	Low complexity (A)	PLS_LC1/PLS_LC2	Extended statement	Structured	Effect size+qualitative statement
8	Low complexity (A)	PLS_LC1/PLS_LC2	Extended statement	Unstructured	Effect size+qualitative statement
9	Low complexity (A)	PLS_LC1/PLS_LC2	Regular statement	Structured	Effect size+glossary
10	Low complexity (A)	PLS_LC1/PLS_LC2	Regular statement	Unstructured	Effect size+glossary
11	Low complexity (A)	PLS_LC1/PLS_LC2	Extended statement	Structured	Effect size+glossary
12	Low complexity (A)	PLS_LC1/PLS_LC2	Extended statement	Unstructured	Effect size+glossary
13	Low complexity (A)	PLS_LC1/PLS_LC2	Regular statement	Structured	Effect size+glossary+qualitative statement
14	Low complexity (A)	PLS_LC1/PLS_LC2	Regular statement	Unstructured	Effect size+glossary+qualitative statement
15	Low complexity (A)	PLS_LC1/PLS_LC2	Extended statement	Structured	Effect size+glossary+qualitative statement
16	Low complexity (A)	PLS_LC1/PLS_LC2	Extended statement	Unstructured	Effect size+glossary+qualitative statement
17	High complexity (B)	PLS_HC1/PLS_HC2	Regular statement	Structured	Qualitative statement
18	High complexity (B)	PLS_HC1/PLS_HC2	Regular statement	Unstructured	Qualitative statement
19	High complexity (B)	PLS_HC1/PLS_HC2	Extended statement	Structured	Qualitative statement
20	High complexity (B)	PLS_HC1/PLS_HC2	Extended statement	Unstructured	Qualitative statement
21	High complexity (B)	PLS_HC1/PLS_HC2	Regular statement	Structured	Effect size+qualitative statement
22	High complexity (B)	PLS_HC1/PLS_HC2	Regular statement	Unstructured	Effect size+qualitative statement
23	High complexity (B)	PLS_HC1/PLS_HC2	Extended statement	Structured	Effect size+qualitative statement
24	High complexity (B)	PLS_HC1/PLS_HC2	Extended statement	Unstructured	Effect size+qualitative statement
25	High complexity (B)	PLS_HC1/PLS_HC2	Regular statement	Structured	Effect size+glossary
26	High complexity (B)	PLS_HC1/PLS_HC2	Regular statement	Unstructured	Effect size+glossary
27	High complexity (B)	PLS_HC1/PLS_HC2	Extended statement	Structured	Effect size+glossary
28	High complexity (B)	PLS_HC1/PLS_HC2	Extended statement	Unstructured	Effect size+glossary
29	High complexity (B)	PLS_HC1/PLS_HC2	Regular statement	Structured	Effect size+glossary+qualitative statement
30	High complexity (B)	PLS_HC1/PLS_HC2	Regular statement	Unstructured	Effect size+glossary+qualitative statement
31	High complexity (B)	PLS_HC1/PLS_HC2	Extended statement	Structured	Effect size+glossary+qualitative statement
32	High complexity (B)	PLS_HC1/PLS_HC2	Extended statement	Unstructured	Effect size+glossary+qualitative statement

PLS, plain language summary; PLS_LC1=PLS of Schwalm et al., 2021; PLS_LC2=PLS of Bucher et al., 2020; PLS_HC1=PLS of Bergmann and Cristia, 2016; and PLS_HC2=PLS of Yule et al., 2019.

It is important to note that the complexity of PLS was not varied within each PLS (i.e., the same PLS was not presented with different “degrees of complexity”). Rather, two entirely different PLS were presented. This approach differs from the procedure for the independent variables statistical terms and structuring, which were varied for each PLS. Consequently, investigating more and less complex PLS constitutes a test of the generalizability of the effects of the other independent variables, which is why all hypotheses were tested in both study arms separately.

#### Procedure

Study procedures were the same as in Study 1. The study was approved by the ethics committee of Trier University. Participants spent a mean duration of approximately 24min on completing the questionnaire.

#### Variables

The same confirmatory outcome variables as in Study 1 were assessed. Because intercorrelations of our outcome measures were very high in Study 1, we made some minor changes to the wording of our confirmatory outcome variables (i.e., we no longer referred to the PLS as “text” but as “summary” and slightly revised our perceived understanding item to stress that this statement aimed at a subjective perception instead of a text characteristic; “I understood the information” instead of “The information is understandable”). Otherwise, the wording and Likert scales of confirmatory outcome variables remained unchanged. Exploratory outcomes and potential covariates assessed for this study are listed in the preregistration of Study 2 materials.

#### Statistical Analysis

##### Sample Size Calculation and Power Analysis

The following parameters were specified in our power analysis for Study 2: Small (*f*=0.10) effect size, alpha=0.05, power=0.875, with four groups (i.e., the maximum number of independent variable conditions realized with regard to the approach for explaining statistical terms), two measures and an expected correlation between measures of 0.50. This power analysis indicated that a sample size of 992 participants was required. Thus, we decided to recruit at least 1,000 participants to test hypotheses 5 and 6. As all hypotheses are tested twice in our study – once for more complex and once for less complex PLS – and additional quota restrictions applied, our target sample size was 2,004 participants.

##### Statistical Model

Study 2 employed the same statistical model and statistical procedures as Study 1. Hypotheses 5, 6, 7, and 8 were tested on the subsample of participants in study arms A and B separately. In study arm A, the reference category for dummy variables was *PLS_LC1* with the *regular statement* for quality of evidence communication, *unstructured* as an approach for structuring and *effect size+statement+glossary* as an approach for explaining statistical terms (i.e., condition 14 in Table 5). In study arm B, the reference category for dummy variables was *PLS_HC1* with the *regular statement* for quality of evidence communication, *unstructured* as an approach for structuring and *effect size+statement+glossary* as an approach for explaining statistical terms (i.e., condition 30 in Table 5). Again, data were discarded if participants failed to provide information on any confirmatory outcomes (i.e., dropped out prior to providing a response, 399 participants) or if participants completed the survey more than once (five participants). Analysis-specific sample sizes are reported in the relevant tables on confirmatory analyses.

### Results

Descriptive statistics on confirmatory outcomes from Study 2 are provided in Table 6. Accessibility, understanding, and empowerment were again highly correlated (all *r*s>0.52). Consequently, we will again report the results of the corresponding confirmatory analyses in an aggregated manner. Mixed model results on Study 2 separated by outcome variable are provided in Tables 7 and 8.

**Table 6:** Study 2 descriptive statistics (means and SDs) of confirmatory outcomes separated by design condition.

Outcome	Approach for explaining statistical terms				Structuring		Quality of evidence communication	
	Effect size+glossary			Effect size+qualitative statement+glossary			Effect size+qualitative statement			Qualitative statement			Structured			Unstructured			Extended statement			Regular statement		
	*M*	*SD*	*n*	*M*	*SD*	*n*	*M*	*SD*	*n*	*M*	*SD*	*n*	*M*	*SD*	*n*	*M*	*SD*	*n*	*M*	*SD*	*n*	*M*	*SD*	*n*
**Low complexity (study arm A)**																								
Accessibility	5.536	1.862	491	5.694	1.844	542	6.125	1.764	601	6.302	1.609	530	5.857	1.882	1,036	5.990	1.711	1,128	5.922	1.765	1,099	5.931	1.829	1,065
Understanding	5.933	1.717	491	5.932	1.836	544	6.240	1.742	601	6.372	1.625	537	6.038	1.804	1,037	6.207	1.680	1,136	6.207	1.699	1,101	6.043	1.782	1,072
Empowerment	4.846	1.751	493	5.029	1.889	547	5.231	1.843	602	5.274	1.676	533	5.005	1.824	1,040	5.194	1.776	1,135	5.175	1.776	1,101	5.030	1.824	1,074
**High complexity (study arm B)**																								
Accessibility	5.433	1.812	573	5.522	1.902	500	6.012	1.707	494	6.133	1.745	533	5.868	1.797	1,074	5.664	1.834	1,026	5.800	1.775	1,072	5.734	1.861	1,028
Understanding	5.822	1.760	573	5.763	1.836	498	6.323	1.574	496	6.247	1.656	531	6.110	1.720	1,072	5.954	1.732	1,026	6.079	1.678	1,068	5.987	1.776	1,030
Empowerment	4.934	1.846	574	4.854	1.946	500	5.253	1.610	499	5.257	1.658	533	5.143	1.785	1,075	4.998	1.771	1,031	5.142	1.741	1,073	5.000	1.816	1,033

*M*=arithmetic mean; *SD*=standard deviation; and *n*=number of observations.

**Table 7:** Study 2 results of confirmatory analyses for user experience outcomes: accessibility, understanding, and empowerment.

Outcome	Parameter	Low complexity (Study arm A)				High complexity (Study arm B)			
		EST	SE	*p*	*R*^2^ Beta	EST	SE	*p*	*R*^2^ Beta
Accessibility Low complexity: *N*_obs_=2,164 *N*_ID_=1,123 High complexity: *N*_obs_=2,100 *N*_ID_=1,085	Random effect (participant) variance	1.738				1.844			
	Residual variance	1.416				1.363			
	Intercept	5.658	0.118	<0.001		5.384	0.126	<0.001	
	Effect size+qualitative statement	0.443	0.130	<0.001	0.008	0.500	0.141	<0.001	0.009
	Effect size+glossary	−0.140	0.137	0.306	0.001	−0.066	0.136	0.630	0.000
	Qualitative statement	0.610	0.134	<0.001	0.014	0.630	0.139	<0.001	0.015
	Contrast 1	−0.583	0.134	<0.001		−0.565	0.137	<0.001	
	Contrast 2	0.167	0.130	0.202		0.130	0.139	0.349	
	Contrast 3	0.750	0.137	<0.001		0.696	0.134	<0.001	
	Quality of evidence extended	−0.020	0.094	0.835	0.000	0.063	0.097	0.520	0.000
	Structured	−0.098	0.095	0.300	0.001	0.197	0.097	0.044	0.003
	PLS_LC2/PLS_HC2	0.139	0.052	0.007	0.002	−0.015	0.051	0.769	0.000
	Marginal/conditional *R*^2^	0.031/0.565				0.032/0.588			
Understanding Low complexity: *N*_obs_=2,173 *N*_ID_=1,121 High complexity: *N*_obs_=2,098 *N*_ID_=1,086	Random effect (participant) variance	1.884				1.760			
	Residual variance	1.121				1.162			
	Intercept	5.861	0.118	<0.001		5.617	0.121	<0.001	
	Effect size+qualitative statement	0.300	0.130	0.021	0.004	0.572	0.136	<0.001	0.013
	Effect size+glossary	−0.005	0.136	0.969	0.000	0.078	0.131	0.555	0.000
	Qualitative statement	0.411	0.133	0.002	0.007	0.507	0.134	<0.001	0.011
	Contrast 1	−0.306	0.133	0.022		−0.494	0.132	<0.001	
	Contrast 2	0.110	0.130	0.396		−0.065	0.134	0.627	
	Contrast 3	0.420	0.137	0.002		0.429	0.129	<0.001	
	Quality of evidence extended	0.155	0.094	0.101	0.002	0.091	0.094	0.332	0.001
	Structured	−0.156	0.094	0.097	0.002	0.168	0.094	0.073	0.002
	PLS_LC2/PLS_HC2	0.136	0.046	0.003	0.002	−0.007	0.048	0.886	0.000
	Marginal/conditional *R*^2^	0.017/0.633				0.024/0.612			
Empowerment Low complexity: *N*_obs_=2,175 *N*_ID_=1,123 High complexity: *N*_obs_=2,106 *N*_ID_=1,086	Random effect (participant) variance	1.952				1.919			
	Residual variance	1.262				1.210			
	Intercept	4.987	0.121	<0.001		4.704	0.125	<0.001	
	Effect size+qualitative statement	0.189	0.133	0.156	0.001	0.404	0.141	0.004	0.006
	Effect size+glossary	−0.182	0.140	0.193	0.001	0.098	0.136	0.475	0.000
	Qualitative statement	0.221	0.137	0.107	0.002	0.405	0.138	0.003	0.006
	Contrast 1	−0.371	0.137	0.007		−0.306	0.136	0.025	
	Contrast 2	0.031	0.137	0.814		0.001	0.139	0.992	
	Contrast 3	0.403	0.141	0.004		0.308	0.134	0.022	
	Quality of evidence extended	0.132	0.097	0.196	0.001	0.147	0.097	0.130	0.002
	Structured	−0.174	0.097	0.072	0.003	0.149	0.097	0.126	0.002
	PLS_LC2/PLS_HC2	0.123	0.049	0.012	0.001	−0.024	0.048	0.624	0.000
	Marginal/conditional *R*^2^	0.013/0.613				0.014/0.618			

Estimates are based on mixed models with (contrasts of) fixed effects for independent variables/PLS and random effects for participants. Separate models were estimated per outcome for PLS with low and high complexity. EST, estimates for variances of residuals and random effects, unstandardized regression weights, and marginal/conditional R-squares (Nakagawa et al., 2013); SE, standard error, *p*=*p*-value of two-tailed significance test (please note that some tests of hypotheses were one-tailed); PLS, plain language summary; *N*_obs_=number of rated PLS; *N*_ID_=number of participants who rated at least one PLS. Intercept=Effect size+statement+glossary, unstructured, regular quality of evidence statement (for PLS with low complexity: PLS_LC1, for PLS with high complexity: PLS_HC1); Contrast 1=(Effect size+glossary) – (Effect size+qualitative statement); Contrast 2=Qualitative statement – (Effect size+qualitative statement); Contrast 3=Qualitative statement – (Effect size+glossary), PLS_LC1=PLS of Schwalm et al., 2021; PLS_LC2=PLS of Bucher et al., 2020; PLS_HC1=PLS of Bergmann and Cristia, 2016; and PLS_HC2=PLS of Yule et al., 2019.

**Table 8:** Study 2 results of confirmatory analyses for content-related knowledge acquisition.

Outcome	Parameter	Low complexity (Study arm A)				High complexity (Study arm B)			
		EST	SE	*p*	OR	EST	SE	*p*	OR
Content knowledge Low complexity: *N*_obs_=2,164 *N*_ID_=1,123 High complexity: *N*_obs_=2,100 *N*_ID_=1,085	Random effect (participant) variance	3.877				1.514			
	Intercept	1.286	0.243	<0.001	3.618	0.682	0.178	<0.001	1.977
	Effect size+qualitative statement	0.175	0.247	0.477	1.192	0.401	0.192	0.037	1.493
	Effect size+glossary	0.085	0.258	0.743	1.088	0.441	0.186	0.018	1.554
	Qualitative statement	0.300	0.255	0.239	1.350	0.448	0.190	0.018	1.566
	Contrast 1	−0.091	0.255	0.722	0.913	0.040	0.188	0.831	1.041
	Contrast 2	0.125	0.250	0.618	1.133	0.047	0.192	0.805	1.048
	Contrast 3	0.216	0.263	0.412	1.241	0.007	0.186	0.969	1.007
	Quality of evidence extended	0.323	0.180	0.074	1.381	−0.323	0.133	0.016	0.724
	Structured	−0.129	0.180	0.475	0.879	−0.019	0.133	0.884	0.981
	PLS_LC2/PLS_HC2	0.541	0.127	<0.001	1.718	0.277	0.108	0.010	1.319

Estimates are based on logistic mixed models with (contrasts of) fixed effects for independent variables/PLS and random effects for participants. Separate models were estimated for PLS with low and high complexity. EST=estimates for variances of residuals and random effects, unstandardized regression weights; SE, standard error, *p*=*p*-value of two-tailed significance test (please note that some tests of hypotheses were one-tailed); OR, odds ratio; PLS, plain language summary. *N*_obs_=number of rated PLS; *N*_ID_=number of participants who rated at least one PLS. Intercept=Effect size+statement+glossary, unstructured, regular quality of evidence statement (for PLS with low complexity: PLS_LC1, for PLS with high complexity: PLS_HC1); Contrast 1=(Effect size+glossary) – (Effect size+qualitative statement); Contrast 2=Qualitative statement – (Effect size+qualitative statement); Contrast 3=Qualitative statement – (Effect size+glossary), PLS_LC1=PLS of Schwalm et al., 2021; PLS_LC2=PLS of Bucher et al., 2020; PLS_HC1=PLS of Bergmann and Cristia, 2016; and PLS_HC2=PLS of Yule et al., 2019.

#### H5 and H6 Approach for Explaining Statistical Terms

##### Accessibility, Understanding, and Empowerment

**Figure 3:**
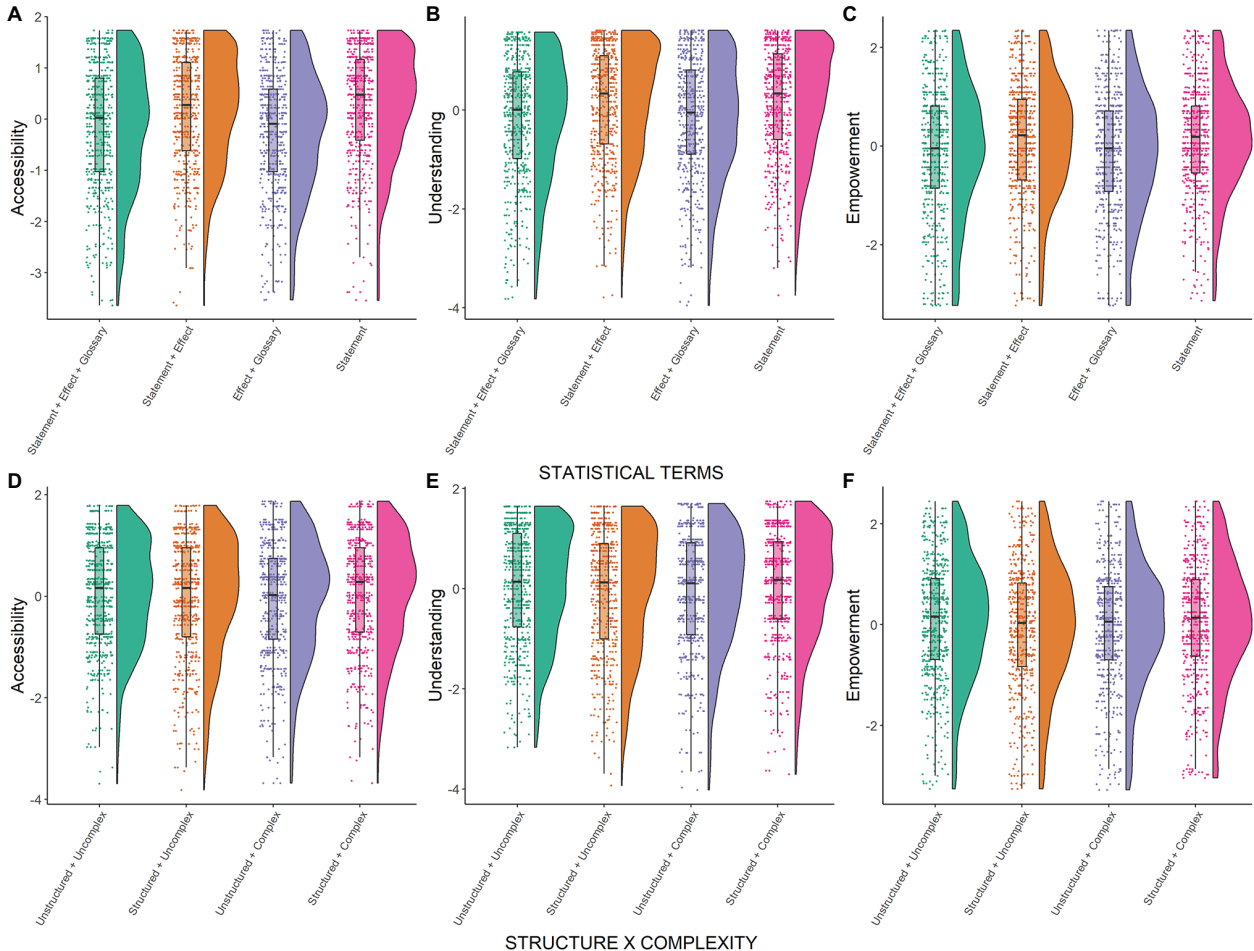
Results study 2. Raincloud plots for user experience outcomes: accessibility **(A,D)**, understanding **(B,E)**, and empowerment **(C,F)**. Residual scores are depicted separated by experimental conditions: Statistical terms **(A–C)**, interaction between structuring and complexity **(D–F)**. Residual scores were obtained from a mixed model that controlled for participant/presented PLS as random factors and for the other independent variables as well as their interactions with complexity as fixed factors.

Descriptively, mean values for user experience outcomes (accessibility, understanding, and empowerment) indicated that participants tended to agree to the corresponding statements (mean values higher than 4.50, see Table 6). In both conditions including a glossary of statistical terms (i.e., *effect size+glossary+qualitative statement*, *effect size+glossary*), user experience was lower compared to conditions providing no glossary (*effect size+qualitative statement*, *qualitative statement*; see Table 6; Figures 3A–C). Inferential analyses revealed that almost all differences were statistically significant (see Table 7). The only exception was that differences for empowerment in PLS with low complexity were non-significant for comparisons involving the *effect size+glossary+qualitative statement* condition (both *p*>0.107, see Table 7). Based on these results, H5a, H5b, H5d, and H6a, H6b and H6d are rejected. Combining a qualitative statement and a glossary in the *effect size+glossary+qualitative statement* condition clearly had no beneficial effects. Finally, throughout all analyses, we observed no significant differences between both conditions that provided no glossary (*effect size+qualitative statement* vs. *qualitative statement*, see Table 7).

##### Content-Related Knowledge

For PLS with high complexity, the proportion of correct answers was descriptively lower in the *effect size+glossary+qualitative statement* condition (61.82%) compared to the *effect size+glossary* condition (69.53%), the *effect size+qualitative statement* condition (68.55%), or the *qualitative statement* condition (69.26%). For PLS with low complexity, the proportion of correct answers was generally higher than for PLS with high complexity, and differences in the proportion of correct answers were less pronounced on a descriptive level (*effect size+glossary+qualitative statement* condition 72.38%, *effect size+glossary* condition 73.17%, *effect size+qualitative statement* condition 74.17%, and *qualitative statement* condition 76.43%). Inferential analyses of our data on PLS with high complexity revealed that the likelihood to answer knowledge items correctly was significantly lower when a qualitative statement combined with a glossary was provided compared to all other conditions (i.e., in the *effect size+glossary+qualitative statement* condition, see Table 8)^6^. There were no significant differences between the other conditions for PLS with high complexity (all *p*s>0.800, see Table 8). For PLS with low complexity, there were no significant differences between all conditions (all *p*s>0.238, see Table 8). Thus, H5c and H6c are rejected as well.

#### H7 Structuring

##### Accessibility, Understanding, and Empowerment

For PLS with high complexity, user experience mean values were descriptively higher for *structured* PLS compared to *unstructured* PLS (see Table 6). The corresponding effect of structuring our PLS was significant for accessibility and understanding, but not for empowerment (see Table 7; Figures 3D–F). For PLS with low complexity, the opposite was true on a descriptive level. Contrary to our hypotheses, user experience mean values were lower for *structured* PLS compared to *unstructured* PLS (see Table 6). Corresponding two-sided inferential tests revealed that these differences were not significant (*p*=0.072–0.300, see Table 7). In sum, H7a and H7b are confirmed for PLS with high complexity only, while H7a and H7b are rejected for PLS with low complexity and H7d is fully rejected.

##### Content-Related Knowledge

For PLS with high and low complexity, the proportion of correct answers was virtually the same for *structured* (more complex=67.07%, less complex=73.20%) and *unstructured* PLS (more complex=67.75%, less complex=74.80%). Inferential analyses revealed that these differences, which were far from significant, did not point in the direction that was proposed in H7c (see Table 8). Thus, H7c is rejected.

#### H8 Extended Quality of Evidence Statement

##### Accessibility, Understanding, and Empowerment

Descriptively, user experience mean values were higher for the *extended* quality of evidence statement that explained complex meta-analytic designs – with accessibility for less complex PLS as an exception (see Table 6). All effects on user experience of the extension of our quality of evidence statement were, however, non-significant (all *p*s>0.109, see Table 7). Thus, H8a, H8b, and H8d are rejected.

##### Content-Related Knowledge

There was a negative effect of our *extended* quality of evidence statement on content-related knowledge for PLS with high complexity. The proportion of correct answers was significantly lower (see Table 8) when the *extended* statement was provided (64.75%) compared to the *regular* statement (70.14%). The corresponding effect for PLS with low complexity was not significant (see Table 8) but pointed in the opposite direction (*extended*=76.05%, *regular*=71.97%). As a consequence, H8c is partially confirmed.

### Interim Discussion

Our results on approaches for explaining statistical terms in PLS (hypotheses 5 and 6) imply that glossaries of statistical terms negatively affect PLS readers’ user experience, regardless of PLS complexity. This might be due to the fact that additional cognitive effort is required to understand this (technical) information. Furthermore, exploratory evidence suggests that content-related knowledge acquisition might have suffered when a glossary *and* qualitative statements were presented for more complex PLS. Thus, our findings clearly implicate that PLS authors should refrain from using glossaries of statistical terms (see also General Discussion on technical limitations of implementing glossaries). Instead, using qualitative statements with or without reporting the corresponding effect size appears to be more beneficial for laypersons.

Our findings on hypothesis 7 suggest that structuring PLS has comparably small beneficial effects on more complex PLS – yet these findings cannot be generalized to less complex PLS. Contrary to our expectations, structuring descriptively impaired user experience for less complex PLS. However, we suggest that our “high complexity” PLS still set a rather low bar for “complexity” and, by far, do not represent the upper end of the obtainable “complexity continuum.” Possible side effects that we have (descriptively) observed for less complex PLS therefore appear unlikely to occur in practice. Thus, we argue that our results tentatively point toward structuring PLS as a way of improving user experience, which is associated with more benefits than drawbacks – especially in the case of highly complex PLS.

The pattern of effects, we observed with respect to the extension of our quality of evidence statement to explain complex meta-analytical designs was rather unexpected. One may argue, in hindsight, that the potential side effects on content-related knowledge for PLS with high complexity are attributable to the reported moderation being non-significant in one of our PLS with high complexity. Because the extended quality of evidence statement explicitly stated that meta-analyses are able to reveal moderator effects, this might have lured readers of our PLS into expecting that these effects indeed exist in the subsequently presented PLS. If future studies confirm this finding, this implies possible drawbacks of educating the public on the potential of scientific methods. This is because statements using illustrative examples as in our study might lead individuals to expect certain findings from applying certain scientific methods. However, our data clearly do not point toward any beneficial effects of an extended statement on the quality of evidence for communicating complex meta-analytic designs in terms of knowledge acquisition or user experience. Consequently, we cannot recommend the wider use of the extended version of this statement.

## General Discussion

### Main Findings

What lessons have we learned so far? As the overarching aim of our research was to develop guidelines for writing PLS, we will summarize our main findings in the form of brief writing instructions before discussing implications, strengths, and limitations of our two studies. The underlying rationale of these instructions – regarding specific statistical results and our hypotheses – can be found in the interim discussion sections of Study 1 and Study 2.

1. Do *not* provide too extensive information on the operationalization of synthesized studies, as this might negatively affect user experience.

However, corresponding effect sizes were very small and providing this additional information did not significantly diminish acquired knowledge. This might indicate that side effects on user experience can be tolerated if you are convinced that providing information on the operationalization is essential for your study.

2. Replace technical terms by non-technical terms if you want to support your audience in grasping the key message of your research.

Providing information on technical terms in a glossary had beneficial effects on user experience in Study 1, where we observed small effect sizes in the glossary and the replacement condition. Side effects of explaining technical terms in a glossary seem to exist, however, in terms of diminished knowledge acquisition. As these side effects showed very small to small effect sizes only, exceptions to this rule might still be appropriate if authors aim to specifically educate their readers about technical terms and concepts. In this specific case, providing a glossary with non-technical expressions might prove worthwhile. However, additional studies explicitly testing PLS effects on the acquisition of the corresponding conceptual knowledge (i.e., do readers really learn the meaning of technical terms) are required to substantiate this claim. We further argue that replacing technical terms might be easier and feel more natural for researchers when writing PLS in their native language (e.g., Cochrane provides PLS in languages other than English, see Kadic et al., 2016; Jakus et al., 2021). Because many technical terms stem from English as the *lingua franca* of science, no direct translations exist for some technical terms in other languages, which is why researchers from non-English speaking countries might automatically replace technical terms by non-technical ones when writing in their native language.

3. Provide information on the quality of evidence of meta-analyses (i.e., an explanation of meta-analyses as a methodological approach) when writing PLS of meta-analyses.

Providing this additional background information will allow audiences unfamiliar with the distinction between primary research and research syntheses to adequately grasp the strengths of claims brought forward in the meta-analysis you summarize. Corresponding effect sizes were very small to small. Moreover, utilizing such an explanatory statement appeared to have no significant negative effects on user experience and knowledge acquisition in our first study. However, findings of our second study suggest that this information should not be too extensive.

4. Carefully consider the amount and type of information on statistical terms you want to provide. If you do not specifically aim at statistically educating your audience, merely provide information on interpreting the effect size without additional details.

Our data indicate that glossaries on statistical terms come at the expense of user experience (with small effect sizes for different approaches on dealing with statistical terms). However, the way in which our glossaries (on technical terms in Study 1 and on statistical terms in Study 2) were presented might have impaired their effectiveness. Providing these texts embedded in websites or as infographics might be more appealing to laypersons (see Strengths, Implications, and Limitations).

5. Formally structure your PLS – especially when they are complex – by means of bullet points and/or use boldface text to highlight key words, if possible.

Our results show that, for PLS with high complexity, the application of formal text structuring improved user experience. Corresponding effects were, however, very small in our study and an exception to this rule exists: Structuring seems to be unnecessary for simple PLS – for example, when PLS focus on only one research finding and a very limited number of theoretical constructs.

### Strengths, Implications, and Limitations

A major strength of our research – especially compared to previous studies on PLS – is that we investigated a total of eight PLS stemming from various psychological (sub-)disciplines. Thus, we are confident that our effects can be generalized to PLS of psychological meta-analyses in general and do not only apply to the specific PLS we studied. Our studies also make a valuable contribution to the existing body of research on PLS by experimentally investigating PLS characteristics in samples that were heterogeneous with regard to age, gender, and educational background (i.e., were not professional or student samples). Overall, we analyzed data of almost 4,500 participants. Thus, we are also quite confident that PLS complying with our rules will appeal to a large and diverse population. Another advantage of the large samples, we recruited is that the power of our analyses for detecting even small effects was high (Study 1: 0.900, Study 2: 0.875). As a consequence, non-significant study findings can be interpreted as evidence of an absence of effects with some degree of certainty.

Findings of our studies also fit in well with broader psychological theories on instructional design – especially cognitive load theory (Sweller, 1994, 2011; Orru and Longo, 2019) – and therefore imply that concepts from cognitive load theory should be considered when drafting PLS for psychological meta-analyses. At its core, cognitive load theory describes relationships between task aspects and the cognitive resources (i.e., working memory capacity) required by those tasks. *Intrinsic cognitive load* relates to the inherent difficulty of a task, whereas *extraneous cognitive load* is caused by other non-inherent factors, such as the presentation mode of a task. It is important that all types of cognitive load draw on the same limited working memory capacity (Engle, 2002). Thus, extraneous load should be minimized to unlock cognitive resources for learning processes, which generate *germane cognitive load*. In Study 1, we observed a positive interaction between interest in psychological topics and providing additional information on operationalization on some outcome variables, as well as a lower knowledge acquisition when a glossary on technical terms was provided. In Study 2, providing additional information *via* a glossary of statistical terms had a negative impact on user experience for PLS with high complexity. Additionally, for complex PLS, participants showed poorer content-related knowledge when receiving an extended quality of evidence statement. Based on cognitive load theory, one might argue that providing additional information on statistics, technical terms or the quality of evidence increases task-relevant intrinsic load (i.e., they make the task of reading and understanding the PLS more difficult) and that this is especially true for readers with low levels of interest or prior psychological concept knowledge. Furthermore, glossaries were presented as separate materials at the end of the respective PLS. This, contrary to a more integrated approach within the PLS itself, may have forced readers to switch between different text passages to mentally integrate information, thereby substantially increasing extrinsic cognitive load and hindering learning processes (see also split-attention effect, e.g., Schroeder and Cenkci, 2018, and contiguity principle, e.g., Mayer and Fiorella, 2014).

Nonetheless, our study is not without some limitations. With regard to our recommendation to leave out glossaries on statistical and technical terms, one might object that educating the public about research findings *via* PLS explicitly includes fostering public understanding of scientific jargon or statistical terms. Starting from this point, replacing technical jargon with non-technical terms or leaving out statistical information do not seem to be feasible approaches. One could argue instead that if one PLS aim entails enhancing knowledge of technical and statistical terms, glossaries (or different types of explanations) should be provided. The same argument could be made for providing information on operationalization, and our knowledge items were possibly limited in this regard as they solely focused on knowledge about the key message of the PLS. This underlines the need for a more nuanced assessment of knowledge gains in future studies on PLS as well as the necessity for individual PLS authors to reflect on specific aims they want to achieve by writing PLS.

Moreover, the way in which, we presented our glossaries on technical and statistical terms might have affected their impact. Participants might prefer explanations to be directly linked to the explicated terms (e.g., by means of pop-up windows) instead of an attached glossary at the end of the PLS. However, this kind of sophisticated approach requires appropriate software solutions which, in turn, might hinder the integration of the corresponding PLS into established reference databases (such as PubMed, PsycInfo, Psyndex, Google Scholar, and Web of Science). The same might hold true for our approaches on structuring PLS. Most journal abstracts consist of non-formatted text, and reference databases might not be able to add bullet points or bold text to text-based objects. Therefore, it might be an encouraging finding that the impact of structuring PLS was comparably small. Nonetheless, addressing these issues in future empirical research, and also from a technical infrastructure perspective, would be highly desirable.

To assess our outcomes, we employed single item measures to put as little strain as possible on our educationally diverse sample in a repeated-measures design. From a psychometric perspective, this is certainly a limitation. There is indeed an urgent need to develop empirically validated measurement tools for scientific research in educationally diverse layperson samples and to translate such tools to languages other than English. In the same vein, we tried to improve the wording of our user experience items in Study 2, but the correlations between accessibility, understanding, and empowerment were still high.

Using eight PLS from various topics might strengthen the generalizability of our findings. There are, however, certain costs of this procedure that become evident when interpreting our results because topic and study arm are confounded. For example, participants did not directly compare two versions of the same PLS with varying complexity in Study 2; rather PLS on different topics were presented here. Especially in Study 2, this makes it hard to interpret differences in the perception of PLS with high complexity and low complexity. Future studies should experimentally vary how many and what kind of effects are reported in a PLS to address this issue. One might also test whether our findings of the first study can be replicated for PLS on other topics or if PLS of the study arms are “switched” (i.e., when information on operationalization is provided for resilience research).

Finally, we acknowledge that many more PLS characteristics exist that are not yet empirically-validated and should be investigated in future research studies. We will refer to our review on PLS (Stoll et al., 2021; see also above) to illustrate this point. In this review, we identified six broad categories of PLS characteristics: linguistic attributes, formal attributes, general content, presentation of results, presentation of quality of evidence, and contextual attributes. In the studies reported in this article, we addressed characteristics of most of these categories: We outlined linguistic attributes as dealing with technical terms, formal attributes as structuring PLS, general content as inclusion of information on the operationalization in synthesized studies or glossaries, presentation of results as approaches for dealing with statistical terms and the presentation of the quality of evidence *via* introductory statements on the meta-analytical approach. Although, we covered a broad range of categories, it is easy to see that we only investigated some isolated (but important) aspects within these broad categories. Because no comprehensive framework on discipline-specific aspects of the lay-friendly communication of psychological science exists, the selection of these investigated characteristics in our studies certainly involved some degree of subjectivity. However, our selection is based on thorough theoretical reasoning, as we followed a comprehensive approach to (1) investigate such aspects that were reflected as core characteristics in our systematic review and (2) investigate those aspects that constitute the core elements (Shneider, 2009) of psychology as a scientific discipline. Not least, in a rather pragmatic approach, we selected such characteristics of which criteria could be readily derived and summarized in a writing guideline. Nonetheless, more work is needed to achieve the aim of empirically validated guidelines on writing psychological PLS.

### Conclusion

Drawing on strong samples and a diverse set of PLS of psychological meta-analyses, our research demonstrates that the way we implement PLS characteristics (i.e., how we write PLS) affects PLS readers’ knowledge acquisition and user experience. Based on the results of two randomized controlled studies, we derived five simple rules that will hopefully support PLS authors in significantly increasing user experience and information uptake in educationally diverse samples. Returning to the basic notion with which we started this article, we hope that the research presented in this article will be valuable to a broad audience. First and foremost, we hope that this article is useful as a means of raising psychological researchers’ awareness of PLS as a tool for transferring psychological evidence to the public and that its findings will maximize the beneficial impact of PLS in psychology. Sticking to the rationale underlying PLS, we, however, also hope that this research will prove to be valuable – beyond this initial target audience – for PLS authors from all disciplines of science.^7^

## Data Availability Statement

Data and code for this publication are available at PsychArchives: data (http://dx.doi.org/10.23668/psycharchives.5211), code (http://dx.doi.org/10.23668/psycharchives.5210).

## Ethics Statement

The studies involving human participants were reviewed and approved by the ethics committee of Trier University. The participants provided their written informed consent to participate in this study.

## Author Contributions

AC, MK, and MS contributed to conception and design of Study 1. All authors contributed to the conception and design of Study 2. MK managed the data, performed the statistical analysis, and wrote the first draft of the manuscript. AC, GB, MJ, and MS wrote sections of the manuscript. All authors contributed to the article and approved the submitted version.

## Conflict of Interest

The authors declare that the research was conducted in the absence of any commercial or financial relationships that could be construed as a conflict of interest.

## Publisher’s Note

All claims expressed in this article are solely those of the authors and do not necessarily represent those of their affiliated organizations, or those of the publisher, the editors and the reviewers. Any product that may be evaluated in this article, or claim that may be made by its manufacturer, is not guaranteed or endorsed by the publisher.

## Footnotes

^1^The numbering of hypotheses 5–8 deviates from the numbering in the corresponding preregistration (hypotheses 1, 2, 3, and 4) because we opted to consecutively number hypotheses throughout this article to avoid any potential confusion.

^2^Since we observed detrimental effects of our operationalization statement in Study 1 (see below), one could, however, argue that presenting this extended quality of evidence statement also increases the complexity of the relevant task, which is why we pre-specified no strict assumption on the direction of effects for hypothesis 8.

^3^ http://dx.doi.org/10.23668/psycharchives.4471

^4^To stay closely in line with our power analysis, we also provide additional ANOVA statistics on these mixed models in Supplementary Material 2.

^5^ http://dx.doi.org/10.23668/psycharchives.4791

^6^This exploratory finding was, however, non-significant when applying the Benjamini-Hochberg correction for multiple testing, with *p* values ranging from 0.055 to 0.074.

^7^For this reason – and to set a good example – we wrote our own PLS for this article which is available as Supplementary Material 4.

## Supplementary Material

The Supplementary Material 1 and 4 for this article can be found online at: https://www.frontiersin.org/articles/10.3389/fpsyg.2021.771399/full#supplementary-material

Supplementary Material 1 Translations of study materials.

Supplementary Material 2 R-Markdown Files: http://dx.doi.org/10.23668/psycharchives.5210.

Supplementary Material 3 Datasets and Codebooks: http://dx.doi.org/10.23668/psycharchives.5210.

Supplementary Material 4 Plain language summary of this article.
