# Supplementary material for: How to Put It Plainly? Findings From Two Randomized Controlled Studies on Writing Plain Language Summaries for Psychological Meta-Analyses
Source: Front Psychol. 2021 Dec 16;12:771399. doi: 10.3389/fpsyg.2021.771399 (PMC8717946; doi:10.3389/fpsyg.2021.771399)
Supplement: Supplementary Material 1 — Translations of study materials. [file Data_Sheet_1.PDF]

Supplemental Material 1. Translations of Study Materials

Table of Contents

1 Study 1 and 2. Statement for communicating the quality of evidence 2

1.1 Quality of evidence statement in Study 1, regular statement in Study 2 2

1.2 Extended quality of evidence statement in Study 2 2

1.3 Knowledge of the quality of evidence in general 3

2 Study 1. Exemplary translations of PLS and PLS-specific items 4

2.1 PLS_RR1: Darling Rasmussen et al. 2019 4

2.1.1 Exemplary non-validated translation of the PLS in the *glossary* condition on technical terms 4

2.1.2 Content-related knowledge 5

2.1.3 PLS-specific knowledge of the quality of evidence 5

2.2 PLS_ID2: Dunst, Gorman and Hamby 2012 6

2.2.1 Exemplary non-validated translation of the PLS in the information *provided* condition on operationalization 6

2.2.2 Content-related knowledge 7

2.2.3 PLS-specific knowledge of the quality of evidence 7

3 Study 2. Exemplary translations of PLS_HC2: Yule, Houston and Grych 2019; and PLS-specific items 8

3.1 Exemplary non-validated translation of the PLS in the *effect size + glossary + qualitative statement* condition on statistical terms and the *structured* text format condition on structuring......8

3.2 Content-related knowledge 10

3.3 PLS-specific knowledge of the quality of evidence 10

1 Study 1 and 2. Statement for communicating the quality of evidence[1]

1.1 Quality of evidence statement in Study 1, regular statement in Study 2

Non-validated translation of the statement on the quality of evidence (Study 1, Study 2: regular statement)

The following statement on the quality of evidence was presented to explain the methodological approach "meta-analysis":

Good to know:

The reviews you just read are meta-analyses. At first, researchers who do a meta-analysis look for the results of the studies on a particular issue (e.g., "How well does a particular psychotherapy help?"). Then they summarize the results of those studies.

Why do they do this? A meta-analysis has two goals:

1) The meta-analysis provides an overview of all studies that have already examined that specific issue. It describes who has already done research on a particular question (e.g., who has already studied this psychotherapy). Also, it describes what was found in each study (e.g., whether psychotherapy helped and how well it helped).

2) The meta-analysis also provides a value that summarizes the results of all studies. To do so, researchers take the individual results of all studies found and calculate an overall result. Therefore, it is important that the studies found on a topic are quite similar. If the studies are very different from each other, the calculated overall result of the meta-analysis will be inaccurate. The calculated overall result provides the researchers with the answer to their question posed at the beginning (e.g., "Across all studies, psychotherapy helps very well."). ***This overall result has a much higher informative value than the results of the individual studies.***

1.2 Extended quality of evidence statement in Study 2

Non-validated translation of the extension of the statement for communicating complex meta-analytic designs only presented in the extended statement condition in Study 2

The following extension of the statement on the quality of evidence was presented in the extended statement condition of Study 2:

In some cases, researchers can even calculate new values out of the data (e.g., how well psychotherapy specifically helps young adults or specifically helps older adults). Then they can use these new values to make new calculations that were not possible before (e.g., whether psychotherapy helps young adults better than older adults). Often, individual studies are too small to allow such calculations because they do not have enough data. So this is another advantage of a

[1] It is not guaranteed that the non-validated English translation corresponds exactly to the language level/proficiency of the German original. Original study materials are available at http://dx.doi.org/10.23668/psycharchives.4791.

meta-analysis: by having a large amount of data, it is possible to examine interrelationships that cannot be examined in individual studies.

1.3 Knowledge of the quality of evidence in general

Non-validated translation of the item to test knowledge on the meaning of the methodological approach “meta-analysis” across all PLS:

"What is a meta-analysis?"

- *A meta-analysis deals with the meta-level of psychology (e.g., intelligence).*
- *A meta-analysis takes place if there are more than 20 researchers in a research group.*
- *A meta-analysis takes place if researchers from different institutions work together.*
- *A meta-analysis deals with the borderline between psychology and metaphysics.*
- *A meta-analysis is an experiment comparing two groups.*
- *A meta-analysis searches for all studies on a research question and summarizes their results.*

2 Study 1. Exemplary translations of PLS and PLS-specific items[2]

2.1 PLS_RR1: Darling Rasmussen et al. 2019

2.1.1 Exemplary non-validated translation of the PLS in the *glossary* condition on technical terms

Attachment Behavior and Resilience

This summary refers to the 2019 review titled "Attachment as a Core Feature of Resilience: A Systematic Review and Meta-Analysis." by P. Darling Rasmussen and colleagues.

What was the aim of this review?

It is assumed that there is a relationship between secure attachment behavior and resilience. This review investigated this relationship.

Key messages:

The review shows that securely attached people tend to be more resilient.

What was studied in the review?

The researchers looked for studies that examined the relationship between attachment and resilience. The researchers found 10 studies of which results were combined in a meta-analysis. Within the studies, attachment behavior was subdivided into secure attachment, insecure-avoidant attachment, and insecure-ambivalent attachment. Then, they looked at the relationship between secure attachment behaviors and resilience across all 10 studies.

What are the main results?

Across the 10 studies, securely attached individuals tended to be more resilient. The correlation was r = .30. This means, the association was weak to moderately strong.

Key terms (The inclusion of this glossary on technical terms was experimentally varied.)

Attachment:

Attachment is the emotional connection with another person. Attachment behavior is what can be observed when people interact with other people. Especially when those people are important to them. Examples of attachment behaviors are smiling or touching someone. People differ in their attachment behavior towards other people.

Attachment Types:

---

[2] It is not guaranteed that the non-validated English translation corresponds exactly to the language level/proficiency of the German original. Original study materials are available at http://dx.doi.org/10.23668/psycharchives.4471.

Three attachment types are often distinguished: secure attachment, insecure-avoidant attachment, and insecure-ambivalent attachment. For example, the attachment type becomes apparent if babies are separated from an important person: Securely attached babies show their feelings if separated and seek contact with the significant other if this person returns. Babies with insecure-avoidant attachment seem to be unimpressed by separation from their caregiver and ignore the returning of this person. Babies with insecure-ambivalent attachment are very upset if separated from the caregiver, and if the caregiver returns, they have difficulties in calming down and are really affectionate.

<u>*Resilience:*</u>

Resilience is a form of inner resistibility. A resilient person has the ability to recover well from stressful events. Therefore, for example, one may rely on one's own experience or knowledge or on the experience of surrounding people. Also, resilient people recover quickly after stressful phases in their lives.

2.1.2 Content-related knowledge

"Which statement about the review you just read is true?"

- *Securely attached people (who feel safe in relationships), show higher resilience (ability to recover from stressful events).*
- *Securely attached people (who feel safe in relationships), show higher muscular hypertrophy (increase in muscle mass) during sports.*
- *Insecurely attached people (who feel insecure in relationships) show aggressive behavior quickly (get angry faster).*
- *Insecure attached people (who feel insecure in relationships) quickly resign (they stop taking certain actions because they think they cannot achieve their goal).*

2.1.3 PLS-specific knowledge of the quality of evidence

"Which statement about the review you just read is true?"

- *In their review, the researchers conducted 10 different studies on attachment behavior (how safe someone feels in relationships) and resilience (ability to recover from stressful events). The researchers evaluated each individual study.*
- *10 researchers examined the relationship between attachment behavior (how safe one feels in relationships) and resilience (ability to recover from stressful events) in a large experiment and published the results of the study.*
- *The researchers examined the relationship between attachment behavior (how safe one feels in relationships) and resilience (ability to recover from stressful events) of 10 individuals. They summarized the results into one single value.*
- *There already were 10 similar studies on how attachment (how safe one feels in relationships) and resilience (ability to recover from stressful events) are related. The researchers combined the results of these 10 studies into one single value.*

2.2 PLS_ID2: Dunst, Gorman and Hamby 2012

2.2.1 Exemplary non-validated translation of the PLS in the information *provided* condition on operationalization

Do babies like baby talk?

This summary refers to the review titled "Preference for infant-directed speech in preverbal young children" by C. Dunst and colleagues in 2012.

What was the aim of this review?

Adults and parents often use baby talk with children who cannot speak yet. It is thought that adults do this because babies like baby talk better than "normal" speech. This review aimed to examine whether babies actually prefer baby talk.

Key messages:

The review indicates that children who cannot speak yet like baby talk better than normal speech.

What was studied in the review?

The researchers looked for studies that compared how babies respond to baby talk and normal speech. The included studies had to use real words and not made-up words. The researchers found a total of 34 studies of which results were combined in a meta-analysis. The babies included in these studies were 2 to 270 days old. Finally, the researchers looked at whether the babies prefer baby talk or normal speech in each of the 34 studies.

How did the 34 studies test differences between baby talk and normal speech?

(The inclusion of this operationalization statement was experimentally varied.)

In the majority of cases, baby talk or normal speech was recorded and then played back to the babies. It was observed how the babies responded to the recordings. There were three types of speech recordings used in the 34 studies: 1) Naturalistic speech recordings, in which women were recorded speaking to adults or babies. 2) Artificial speech recordings, in which speakers were asked to read aloud given texts in baby talk or adult speech. 3) Computer-generated speech recordings sounding like baby talk or adult speech. Mostly, the speakers were women unknown to the babies. The babies' behavior while hearing the speech was utilized as a measure of preference. Preference was assessed by head movements, gazes directed to the recordings or the babies' mood while listening to the recordings.

What are the main results?

Across the 34 studies babies liked baby talk better. The effect size Cohen's d was 0.67, indicating that the difference was medium-sized. Depending on how the studies examined preference, differences could be found. For example, the difference was larger for natural speech than for artificial or computer-generated speech.

2.2.2 Content-related knowledge

"Which statement about the review you just read is true?"

- *Babies liked baby talk better than normal speech.*
- *Babies did not like baby talk better than normal speech.*
- *Babies liked normal speech better than baby talk.*
- *Babies liked baby talk better than normal speech if it was computer-generated.*

2.2.3 PLS-specific knowledge of the quality of evidence

"Which statement about the review you just read is true?"

- *In their review, the researchers conducted 34 studies on whether babies like baby talk.*
- *There already were 34 similar studies on whether babies like baby talk. The researchers reviewed these and then examined the same question by another study with a slightly different experimental design.*
- *The researchers tested a total of 34 babies to find out whether babies like baby talk.*
- *There were already 34 similar studies on whether babies like baby talk. The researchers combined the results of these 34 studies into one single value.*

3 Study 2. Exemplary translations of PLS_HC2: Yule, Houston and Grych 2019; and PLS-specific items[3]

3.1 Exemplary non-validated translation of the PLS in the *effect size + glossary + qualitative statement* condition on statistical terms and the *structured* text format condition on structuring.[4]

What enables children to better recover from experiences of violence?

This summary refers to the 2019 review titled "Resilience in Children Exposed to Violence: A Meta-analysis of Protective Factors Across Ecological Contexts" by Kristen Yule and colleagues.

What was the aim of this review?

Background: *Some children experience violence in everyday life. Children who experience violence often develop challenging behaviors and mental health problems. However, this is not true for all children who experience violence. Some children develop normally despite experiencing violence. These children can recover particularly well from stressful events.*

Research question: *In the review, the researchers addressed the following research questions: Why do some children have the ability to recover from stressful events and others do not? Which factors are important in processing experiences of violence?*

Key messages:

Five protective factors were found to help children cope with experiences of violence: family support; school support; peer support; involvement in a religious community and the ability to manage one's own feelings and behavior.

What was studied in the review?

What studies were looked for? *The researchers looked for studies that examined the relationship between exposure to violence and the ability to recover from stressful events in minors.*

Which studies were found? *In total, the researchers found 118 studies of which results were combined in a meta-analysis.*

What did the researchers do? *Within the meta-analysis, it was then examined which so-called protective factors were related to a higher ability to recover from stressful events. The protective factors examined were family factors, peer or school support, and personal or social factors. The meta-analysis not only examined whether the associations between experience of violence, protective*

---

[3] It is not guaranteed that the non-validated English translation corresponds exactly to the language level/proficiency of the German original. Original study materials are available at http://dx.doi.org/10.23668/psycharchives.4791.

[4] In the condition with *unstructured* text format, subsections were not split into the paragraphs Background, Research question, What studies were looked for?, Which studies were found?, What did the researchers do?, What was studied?. Furthermore, there were no bullet points for reporting the investigated variables (What was studied?) and results (What are the main results?).

factors, and the ability to recover from stressful events existed at the time of the interview (i.e., currently), but also were evident over time (i.e., in the long term) among the affected children.

What was studied?

- *ability to recover from stressful events (e.g., experience of violence)*
- *family factors*
- *peer support*
- *school support*
- *personal factors*
- *societal factors*
- *time period: current or long-term*

What are the main results?

Across 118 studies, 11 different protective factors were tested. Of these, 5 were particularly important because their protective effects can be found both at present and in the long term.

- *Family support was one of the protective factors. Here, the effect size ranged from R = 0.16 to R = 0.18. This means the association was weak.*
- *Another protective factor was school support. Here, the effect size ranged from R = 0.20 to R = 0.21. This means the association was weak.*
- *Also, peer support turns out to be a protective factor. Here, the effect size was R = 0.12. Again, this is a weak association.*
- *Within the personal factors, the ability to control one's own feelings and behavior was a protective factor. Here, the effect size ranged between R = 0.30 and R = 0.45. This means the association was moderately strong.*
- *A social protective factor was being part of a religious community. Here, the effect size ranged between R = 0.05 and R = 0.16. This means the association was very weak to weak.*

Therefore, these five protective factors may strengthen the ability to recover from stressful events of children who have experienced violence.

The inclusion of the following glossary on statistical terms was experimentally varied.

Weighted effect size R: *Indicates the direction and strength of the association between two characteristics (e.g., duration of therapy and well-being). R values range from -1 to +1. If R is greater than 0, there is a positive association. Then, for example, with a higher number of therapy sessions, well-being continues to increase. If, on the other hand, R is less than 0, then there is a negative association. For example, this is the case if well-being continues to decrease with a higher number of therapy sessions.*
The closer R approximates +1 or -1, the stronger is the positive or negative association. Though, the following rules of thumb can be used:

- *Up to values of R = +/- .10, the association is very weak and negligible*
- *From values of R = +/- .10, the association is weak*
- *From values of R = +/- .30 the association is moderately strong*
- *From values of R = +/- .50 the association is strong*

"Weighted" means that the authors made R comparable across all studies. Thus, it is not important if studies included different numbers of participants.

3.2 Content-related knowledge

"Which statement about the review you just read is true?"

- *Five factors were found to differentiate effective therapies for children who have experienced violence.*
- *Five factors were found helping children to handle experiences of violence.*
- *Five factors were found that differentiate experiences of violence.*
- *Five factors were found that protect children from experiencing violence.*

3.3 PLS-specific knowledge of the quality of evidence

"Which statement about the review you just read is true?"

- *In their review, the researchers conducted 118 different studies on protective factors for children who have experienced violence. The researchers evaluated every single study.*
- *118 researchers examined protective factors for children who have experienced violence in a large experiment and published the results of the study.*
- *The researchers examined protective factors for 118 individuals who have experienced violence. They combined the results into one single value.*
- *There have already been 118 similar studies on protective factors for children who have experienced violence. The researchers summarized the results of these 118 studies.*
