# Supplementary material for: How to Put It Plainly? Findings From Two Randomized Controlled Studies on Writing Plain Language Summaries for Psychological Meta-Analyses
Source: Front Psychol. 2021 Dec 16;12:771399. doi: 10.3389/fpsyg.2021.771399 (PMC8717946; doi:10.3389/fpsyg.2021.771399)
Supplement: Supplementary Material 4 — Plain language summary of this article. [file Data_Sheet_4.pdf]

frontiers

Plain Language Summary

This text is a plain language summary (PLS) of our study with the title "How to put it plainly? Findings from two randomized controlled studies on writing plain language summaries for psychological meta-analyses" from 2021. The authors are Martin Kerwer, Marlene Stoll, Mark Jonas, Gesa Benz, and Anita Chasiotis.

What is the aim of the study?

<u>Background:</u> PLS summarize the findings of a scientific publication for people who are not experts. They explain what the researchers did and what they found out. Experts have not yet agreed on exactly how PLS should be written. That is why research on PLS is needed. The aim of this research is to best support readers in understanding the scientific content of the PLS. In this study, we focus on PLS of psychological meta-analyses. Meta-analyses summarize research findings. Therefore, they are especially important to the public.

<u>Research Questions:</u> What are common sense rules for writing PLS in psychology, specifically for psychological meta-analyses?

Key message of this study

Based on experiments, we developed five rules for writing PLS of psychological meta-analyses. When writing a PLS, authors should:

- … replace scientific jargon with non-technical words.
- … structure the PLS by using subheadings or bullet points.
- … avoid detailed information about how the single studies were carried out.
- … avoid too much statistical information and focus rather on the interpretation of the results.
- … explain what a meta-analysis is.

What was the way of working on this research question?

<u>What did the experiment look like?</u> To find out how PLS should be written, we carried out two online experiments. We wrote different versions of eight PLS on psychological topics. In all, we varied six PLS characteristics. For example, we varied how technical and statistical terms were reported. Each participant read two PLS. Afterwards, they answered questions about how much they liked the PLS, and they filled in a knowledge test.

<u>Who participated?</u> In all, 4,499 people participated in the experiments. They differed in their education level, age and gender. This was important to us since we want our PLS to be understood by as many people as possible.

What are the most important results?

We found that most people preferred a simple replacement of technical and statistical terms in the text. Additionally, explaining how a meta-analysis works helped them to make decisions and to understand what was done. Further formatting of the text by bullet points was only helpful for complex PLS. We also found that our participants did not like PLS that explained how the single studies included in a meta-analysis were carried out. In their opinion, this made the PLS harder to understand.

Why are these results important?

We hope that these rules will support researchers in writing PLS that everyone can understand. Such PLS could help laypersons to use scientific findings for their own benefit in their everyday life.
